# What works for whom in compassion training programs offered to practicing healthcare providers: a realist review

**DOI:** 10.1186/s12909-021-02863-w

**Published:** 2021-08-28

**Authors:** Shane Sinclair, Jane Kondejewski, Priya Jaggi, Amanda L. Roze des Ordons, Aliya Kassam, K. Alix Hayden, Daranne Harris, Thomas F. Hack

**Affiliations:** 1 Faculty of Nursing, University of Calgary, 2500 University Drive NW, Calgary, Alberta T2N 1N4 Canada; 2 Division of Palliative Medicine Department of Oncology, Cumming School of Medicine, University of Calgary, 2500 University Drive NW, Calgary, Alberta T2N 1N4 Canada; 3 Compassion Research Lab, University of Calgary, 2500 University Drive NW, Calgary, Alberta T2N 1N4 Canada; 4 Department of Critical Care Medicine and Division of Palliative Medicine Department of Oncology, Cumming School of Medicine, University of Calgary, Calgary, Alberta Canada; 5 Department of Community Health Sciences, University of Calgary Cumming School of Medicine, Calgary, Alberta Canada; 6 Office of Postgraduate Medical Education, University of Calgary, Cumming School of Medicine, Calgary, Alberta Canada; 7 Libraries and Cultural Resources, University of Calgary, Calgary, Alberta Canada; 8 College of Nursing, Rady Faculty of Health Sciences, University of Manitoba, 99 Curry Place, Winnipeg, Manitoba R3T 2M6 Canada; 9 Psychosocial Oncology & Cancer Nursing Research, St. Boniface Hospital Research Centre, Room CR3018, 369 Taché Ave, Winnipeg, Manitoba R2H 2A6 Canada

**Keywords:** Compassion, Education, Healthcare providers, Training, Clinicians, Nurses

## Abstract

**Background:**

Patients and families want their healthcare to be delivered by healthcare providers that are both competent and compassionate. While compassion training has begun to emerge in healthcare education, there may be factors that facilitate or inhibit the uptake and implementation of training into practice. This review identified the attributes that explain the successes and/or failures of compassion training programs offered to practicing healthcare providers.

**Methods:**

Realist review methodology for knowledge synthesis was used to consider the contexts, mechanisms (resources and reasoning), and outcomes of compassion training for practicing healthcare providers to determine what works, for whom, and in what contexts.

**Results:**

Two thousand nine hundred ninety-one articles underwent title and abstract screening, 53 articles underwent full text review, and data that contributed to the development of a program theory were extracted from 45 articles. Contexts included the clinical setting, healthcare provider characteristics, current state of the healthcare system, and personal factors relevant to individual healthcare providers. Mechanisms included workplace-based programs and participatory interventions that impacted teaching, learning, and the healthcare organization. Contexts were associated with certain mechanisms to effect change in learners’ attitudes, knowledge, skills and behaviors and the clinical process.

**Conclusions:**

In conclusion this realist review determined that compassion training may engender compassionate healthcare practice if it becomes a key component of the infrastructure and vision of healthcare organizations, engages institutional participation, improves leadership at all levels, adopts a multimodal approach, and uses valid measures to assess outcomes.

**Supplementary Information:**

The online version contains supplementary material available at 10.1186/s12909-021-02863-w.

## Background

Compassion, “*a virtuous and intentional response to know a person, to discern their needs and ameliorate their suffering through relational understanding and action”* [1], is considered a hallmark of quality patient care that is lacking from patients’ experiences of healthcare and healthcare providers’ training. While easily dismissed as an ‘optional extra’, compassion is a salient form of care that addresses each of the domains for optimizing health system performance: improving patient outcomes, enhancing the patient experience, increasing healthcare provider well-being, and lowering healthcare costs [2–9]. Emerging evidence suggests that compassion has a positive effect on patient symptoms [10–12], quality-of-life [11, 13–15], and is one as one of the greatest predictors of the patient experience [7, 16–18]. Compassion also has a strong positive effect on healthcare providers by reducing moral distress, burnout and occupational stress, while increasing healthcare provider work engagement, job satisfaction, and retention [19–25]. Sadly, the importance of compassion is most pronounced when it is lacking, with deficiencies in compassion being identified as a core contributor to healthcare delivery failures including adverse patient events, greater numbers of patient/family complaints and malpractice claims, and increased healthcare costs [7, 8, 11, 25–39].

While implementing compassion training for future healthcare providers may seem reasonable [40], compassion is most challenged among practicing healthcare providers who aspire to provide compassion, yet increasingly encounter system-related barriers, such as insurmountable workloads, limited resources, low team morale, and a lack of leadership [1, 41–45]. The need to improve compassion in healthcare has been recognized by governments, healthcare organizations, and healthcare provider licensing bodies following a number of healthcare failures [30, 46–51]. This resulted in an influx of training programs designed to enhance and sustain healthcare providers’ attitudes, knowledge, skills, and behaviors related to compassion. A recent systematic review, detailing the content, methods, and quality of compassion training in healthcare, revealed that these compassion training programs faced significant barriers [52], including, but not limited to, curricula that did not address the multiple domains of the complex construct of compassion [1, 53], challenges associated with implementation, long-term clinical application and sustainability, and inadequate inclusion of patient perspectives.

While the quality of the evidence characterizing compassion training programs is an important consideration in evaluating existing training programs and implementing new programs, it cannot provide solutions to the complex problems associated with implementing these programs in challenging clinical contexts among diverse learners and healthcare teams [54]. A comprehensive understanding of what works, how, why, for whom, to what extent and in what circumstances while taking into account the content, format and process of compassion training is needed. Only then can we accurately implement compassion training for practicing healthcare providers that is tailored to their diversity of learning and specialty needs.

This study used realist review methodology for knowledge synthesis [54, 55] to consider the contexts, mechanisms, and outcomes that contribute to the success or failure of compassion training for practicing healthcare providers.

The objectives of this study were to 1) identify the context, mechanisms, and outcomes (CMOs) of compassion training programs offered to practicing healthcare providers and 2) apply these CMO configurations in explaining the successes and/or failures of these compassion training programs.

## Methods

### Study design

This study was conducted according to the guidelines for conducting and reporting a realist review, provided in the Realist and Meta-narrative Evidence Syntheses – Evolving Standards (RAMESES) [56].

A realist review uses heterogeneous evidence to answer a research question about a complex social intervention with a plausible explanation or theory [54, 55]. The overarching research question for this realist review was: What works, for whom, and in what contexts in relation to compassion training for practicing healthcare providers? To guide the review, this overarching research question was refined to three specific questions:

1. In what *contexts* does compassion training for practicing healthcare providers take place?
2. What are the key *mechanisms* that act as facilitators or barriers to compassion training for practicing healthcare providers?
3. What are the *outcomes* of compassion training in practicing healthcare providers?

Findings were expressed as Contexts (C) + Mechanisms (M) = Outcomes (O). In realist reviews, CMO configurations should be identified during data analysis in relation to the specific intervention being considered [57–59]. In this review, contexts were defined as conditions in which compassion training was introduced and that triggered the training (background circumstances/unmet need); mechanisms explained the impact of the component introduced by the context (the underlying resources) on the cognitive or emotional decisions and behaviors of the learners (reasoning) that caused compassion training to produce a change; and outcomes were defined as intended and/or unintended consequences of compassion training [57–59].

### Sample

Database searches, article review, and data extraction occurred from March to October 2020. The sampling strategy is summarized in Additional file 1.

The review began with a sample of studies (hereafter referred to as the index articles) included in a recent systematic review of the content, methods and quality of compassion training in healthcare [52]. These studies had been identified through systematic searching of the following databases: MEDLINE, Embase, CINAHL, Sociological Abstracts, Web of Science, ERIC, and Education Research Complete from inception to May 2020. The studies developed, conducted, and evaluated compassion training (i.e. curricula, workshops, rounds, education programs, professional development, lectures, seminars, rotations) for practicing healthcare providers with the objective and/or outcome directed at enhancing healthcare provider compassion towards patients and/or families.

The index articles were supplemented with additional records from a CLUSTER search, which was conducted using Web of Science, Google Scholar (via Publish or Perish), Scopus, and the lead author’s Web pages. In CLUSTER searching: Citation identifies records of particular relevance to the review question; Lead authors involves checking publication lists; Unpublished material is identified by contacting lead authors; Scholar searches involves conducting named project and citation searches; Theories are identified by rechecking the reference database for mention of theory; Early examples involves further citation searching to identify antecedent (earlier examples/pilot studies) projects; and Related projects involves further citation searching to identify related contemporary projects [60]. This search included the CLUSTER elements: lead author publications, ‘cited by’ publications, and related publications (limited to the top 20 most relevant, articles and English language). CLUSTER search results were compiled using Covidence software (Veritas Health Innovation, Melbourne, Australia).

### Data extraction and analysis

Methodology relevant to data extraction and analyses followed that of a previous realist review investigating quality improvement curricula in undergraduate and postgraduate medical education [61].

The full texts of the index articles were reviewed independently by three review authors (JK, PJ, DH) for data-driven quantitative or qualitative explanations of why training was more or less successful at promoting the requisite attitudes, knowledge, skills, and behaviors associated with compassion in practicing healthcare providers. A fourth review author (AR) checked a 10% subsample of the index articles. Congruence in data extraction was 90.1%, representing the number of agreements/number of agreements + disagreements [62]. All disagreements were resolved through discussion between reviewers until consensus was reached.

Records identified by the CLUSTER search were screened and reviewed independently by two review authors (JK, PJ). Only records that included descriptive detail or a discussion that contributed to the development of data- or theory-driven explanations about why and how compassion training might work were included.

An instrument designed by the research team was used to extract information from all included records, including details of the compassion training (participants, type of training, duration and frequency, content), contexts, mechanisms, and outcomes (see Additional file 2). The Kirkpatrick model was used to evaluate outcomes. The Kirkpatrick model consists of four levels of evaluation that were designed to appraise training programs, where Level 1 Reaction, considers learners reactions to training; Level 2 Learning, considers the attitudes, knowledge and skills acquired by learners during training; Level 3 Behavior, considers changes in learners’ subsequent behavior after training; and Level 4 Results, considers whether improvements have been made at the clinical level as a result of training [63]. Methodological quality of the index articles was appraised previously using the Cochrane Collaboration’s domain-based evaluation (selection, performance, detection, attrition, reporting, and other biases) [44]. For the purpose of the realist review, the relevance of all included records to theory building, and their rigor, determined based on the experimental or empirical approach used to generate the data, were assessed on a five-point scale, where 1 = no relevance or rigor and 5 = extremely relevant or exceptionally rigorous [61].

Among the index articles, patterns and associations between contexts, mechanisms, and outcomes were explored to develop a preliminary program theory, which was refined using information extracted from records identified by the CLUSTER search.

## Results

**Fig. 1:**
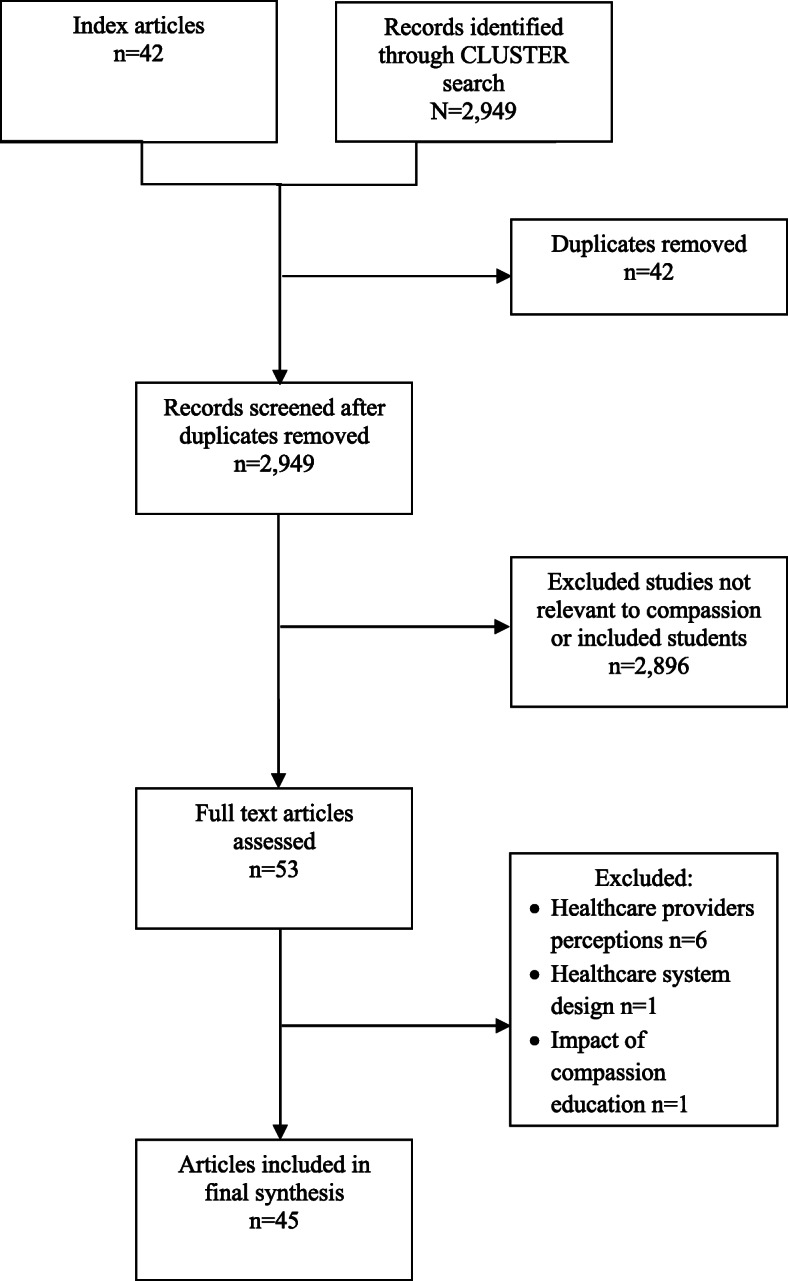
Flow chart of study selection

A total of 42 index articles underwent full-text assessment and contributed to the development of the preliminary program theory. The CLUSTER search identified 2949 records, of which 11 records underwent full text review. As 8 records did not contribute to the refinement of the program theory, 3 records were included in the final synthesis (Fig. 1).

**Fig. 2:**
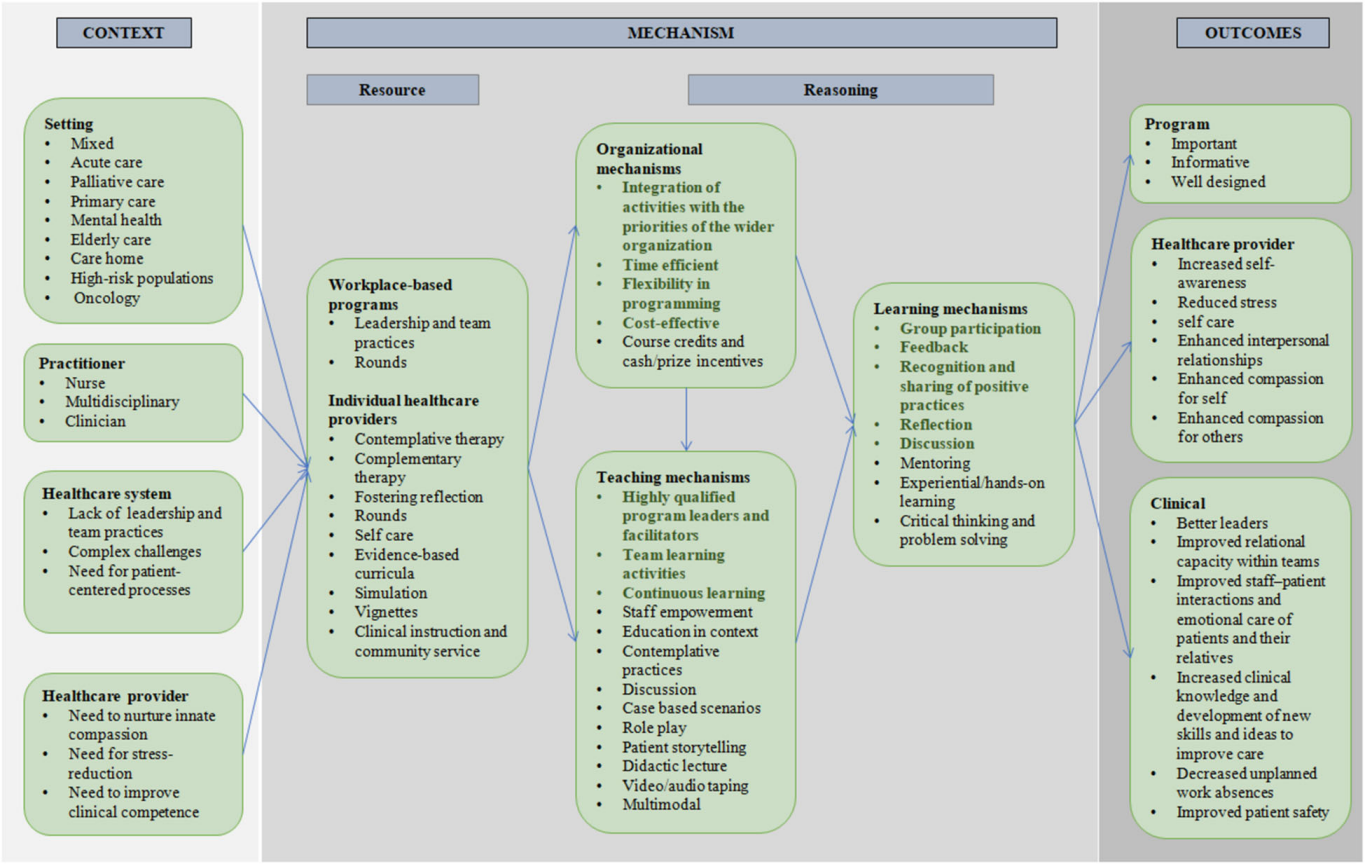
Program theory showing the relationships between contextual factors, mechanisms and outcomes (right) for compassion training. Bolded items have a stronger association with compassion training outcomes. Contexts were defined as conditions in which compassion training was introduced and that triggered the training (background circumstances/ unmet need); mechanisms explained the impact of the component introduced by the context (the under lying resources) on the cognitive or emotional decisions and behaviors of the learners (reasoning) that caused compassion training to produce a change; and outcomes were defined as intended and/or unintended consequences of compassion training (57, 58, 59). *bolded items have a stronger association with compassion training outcomes

The characteristics of all included records are summarized in Table 1 Details of each compassion training program are described in Additional file 3. Summaries of the contexts and mechanisms associated with the outcomes of compassion training for each compassion training program are provided in Additional file 4. All associated references are cited in Additional file 5, and pertinent associated references are cited in the text below. A high-level summary of the contexts and mechanisms associated with the outcomes of compassion training is described in Table 2, while a visual summary of the program theory is provided in Fig. 2.

**Table 1:** Major characteristics of the included records

Study characteristic	No. (%^**a**^) of studies	References
**Geographic location**		
USA	19 (42%)	[64–81]
UK (incl. Scotland and Ireland)	19 (42%)	[18, 81–99]
Canada	1 (2%)	[100]
Netherlands	1 (2%)	[101]
Singapore	1 (2%)	[102]
Germany	1 (2%)	[103]
Mexico	1 (2%)	[104]
New Zealand	1 (2%)	[105]
Other	2 (4%)	[106, 107]
**Training program**		
Curricula	3 (7%)	[75, 78, 104]
Service learning	1 (2%)	[79]
Leadership and team building programs	12 (27%)	[18, 83–89, 94–96, 99]
End of life care		[74, 76, 99]
Specific patient populations	3 (7%)	[75, 77, 78, 90]
Self reflection	5 (11%)	[80, 81, 98, 106]
Clinical simulation	2 (4%)	[94, 97]
Role modeling	2 (4%)	[74, 91]
Contemplative therapy	11 (24%)	[66, 67, 69, 70, 72, 82, 92, 93, 101–103]
Complementary therapy	4 (9%)	[64, 65, 68, 73]
Other	3 (7%)	[71, 100, 105]
**Learner type**		
Nurses	22 (49%)	[18, 65, 70, 73, 74, 76, 77, 79, 83, 85, 86, 89, 91–93, 95, 96, 100]
Nursing professors	2 (4%)	[79, 107]
Critical care nurses	1 (2%)	[75]
Midwives	2 (4%)	[85, 87]
Physicians	4 (9%)	[66, 67, 101, 106]
Palliative physicians	1 (2%)	[80]
Mental health providers	3 (7%)	[82, 97, 102]
Staff^b^	13 (29%)	[64, 68, 69, 71, 72, 78, 81, 88, 90, 94, 98, 99, 103–105]
**Evaluation**		
Self report	34 (76%)	[64–76, 78–82, 87, 90–94, 97, 98, 100–107]
External assessment	1 (2%)	[77]
Self report and external assessment	10 (22%)	[18, 83–86, 88, 89, 95, 96, 99]

^a^Percentages may be > 100% as some studies were included in multiple categories

^b^Unspecified healthcare professionals

**Table 2:** Summary of the contexts, mechanisms, and outcomes most commonly associated with compassion training for practicing healthcare providers

***Context***^a^		***Description of contexts***
Setting		• Mixed, acute care, palliative care, primary care; mental health; elderly care; care home; high-risk populations; oncology
Healthcare provider		• Nurses; clinicians; multidisciplinary
Healthcare system context	Need for an integrated approach to care	• Lack of leadership and team practices that nurture and sustain individual caregiver relational capacity • Creation of unpredictable pressures due to complex challenges in the health-care setting • Need for patient-centered processes aimed at improving efficiency, safety, and the patient experience to be central to the delivery, and evaluation of health care services
Healthcare provider context	Need to nurture innate compassion	• Need to understand the personal and clinical experiences of healthcare providers as they impact the healthcare provider-patient relationship
	Need for stress-reduction	• Stress and burnout impact negatively on the caring relationship and the healing environment
	Need to improve clinical competence	• The specific knowledge, skills and attitudes of healthcare providers must be increased to improve quality of care
***Mechanisms***^***b***^		***Description of mechanism***
Resource	Workplace based learning	• Development of leadership and team practices giving healthcare providers a framework to deliver compassion based on human interactions. • Rounds, multidisciplinary forums in which participants reflect on clinical experiences.
	Participatory interventions supporting individual healthcare providers	• Contemplative therapy, including mindfulness and compassion-oriented practices, meditation, and mind body therapies • Complementary therapy, including Healing Touch (an integrative biofield therapy) and Compassionate Touch as nonpharmacological approaches to patient care • Fostering reflection through bounded time or use of various media such as literature, art, writing and sharing narratives • Rounds • Self care through contemplative and complementary therapies • Evidence-based curricula targeted at specific patient populations • Simulation and role play • Vignettes focused on improving patient and family centred care • Clinical instruction and community service
Reasoning	Teaching mechanisms	• Highly qualified program leaders and facilitators who provided mentoring, role modeling, advice and training • Team learning activities, e.g., developing a community of practice, implementing collective action plans, and conducting local team climate analyses, values clarification, team study days, and team discussions • Continuous learning, e.g. ensuring clinical work covered by float healthcare providers, offering booster sessions, and sending weekly reminders and tips • Staff empowerment • Education in context • Contemplative practices • Discussion, briefing and debriefing following ‘best practice’ • Case based scenarios • Role play • Patient storytelling • Didactic lecture • Video/audio taping • Multimodal
	Learning mechanisms	• Group participation, created a sense of community and a vital common goal • Feedback, generated discussion on why particular interactions worked well • Recognition and sharing of positive practices • Reflection, increased learners subjective understanding of the patient experience • Discussion • Mentoring, provided a safe teaching environment • Experiential/hands-on learning • Critical thinking and problem solving • Linking theory to practice
	Organizational mechanisms	• Integration of activities with the priorities of the wider organization • Time efficient e.g., abbreviated format with dedicated online resources • Flexibility in programming • Cost-effective, costs of compassion training balanced against organizational benefits and cost savings of increasing the quality of care • Course credits and cash/prize incentives
***Outcomes***^***c***^		***Description of outcomes***
	Program level	• Training was perceived to be highly important • Training was informative, engaging and enjoyable • Training was well designed, delivered and facilitated
	Healthcare provider level	• Increased self-awareness • Reduced burnout, depression, anxiety, and stress • Promoted self care • Enhanced interpersonal relationships • Enhanced compassion for self and compassion for others
	Healthcare system level	• Created better leaders who assumed authority, implemented change, and supported team learning • Improved relational capacity within teams • Improved staff–patient interactions and emotional care of patients and their relatives • Increased clinical knowledge and development of new skills and ideas to improve care • Decreased unplanned work absences • Improved patient safety

^a^Contexts were defined as conditions in which compassion training was introduced and that triggered the training (background circumstances/ unmet need); ^b^mechanisms explained the impact of the component introduced by the context (the underlying resources) on the cognitive or emotional decisions and behaviors of the learners (reasoning) that caused compassion training to produce a change; and ^c^outcomes were defined as intended and/or unintended consequences of compassion training (Dalkin et al., 2015; Jolly and Jolly, 2014; Salter and Kothari, 2014)

### Contexts

Contexts in which compassion training for practicing healthcare providers were introduced included the clinical setting, healthcare provider characteristics, current state of the healthcare system, and personal factors that impacted individual healthcare providers.

Many healthcare providers (30%) participating in compassion training were working in a variety of clinical care settings, while others were providing acute (24%), palliative (15%), or primary (8%) care, or working in mental health (8%), elder care (6%), a care home (2%), with high-risk populations (HIV/AIDs; 2%), or oncology (2%). Most healthcare providers participating in compassion training were nurses (53%). Compassion training was also offered across multiple disciplines. Learners were predominantly female, Caucasian, between the ages of 18 and 74 years, with 0–44 years of clinical experience. While leaders were recruited, most other learners participated through voluntary attendance. A few learners were given paid time for training but were resistant as they did not consider it ‘part of their job’ [64]. The majority of learners had no previous experience with compassion training, although some had previously participated in contemplative practices or complementary therapy [64, 65, 82], (details of the learners participating in each compassion training program are provided in Additional file 3 and Additional file 6).

At the healthcare system level, compassion training was predominantly triggered by system-related failures characterized by: poor leadership and team practices [18, 83–87]; a culture that favored high patient throughput over the relational aspects of care [88, 89]; and patient complaints about gaps in the provision of compassionate care [90, 91]. At the individual healthcare provider level, compassion training addressed the need to: nurture innate compassion; reduce learner stress; and improve learner clinical competence. Some learners positioned themselves as already having a compassionate approach [66, 67, 103]. These learners wanted to strengthen their professional proficiency in compassion, believing that conveying compassion was an integral part of their work. Other learners required attitudinal changes to facilitate and maintain the development of compassion. Some learners faced complex stressors in the healthcare setting, such as high workloads, staff shortages, resource constraints, conflicts with coworkers, and meeting the many needs of patients and their families [65, 66, 68–70, 92, 102, 103]. These challenges have been associated with job-related absenteeism, burnout, and high turnover rates, and can impact the delivery of high-quality patient care [66, 67, 71, 72, 93, 101]. Other learners lacked specific skills and knowledge, which reduced clinical engagement with their patients [73–77, 94].

### Mechanisms

#### Resources

Resources implemented to strengthen compassion in the delivery of healthcare included workplace-based programs designed to encourage the development of sustainable high-quality leaders and team relational practices, and participatory interventions designed to enhance individual learners’ professional development, coping, and resilience skills.

Workplace-based programs aimed to promote leadership and team-based practices in the delivery of high-quality compassionate healthcare. These programs attempted to embed a combined set of expansive activities into the workplace [83, 84, 95, 96] or use appreciative inquiry to locate, affirm, leverage and encourage existing organizational assets, capabilities, resources and strengths to support compassionate care [18, 88, 89]. Individual learners’ professional development was enhanced through evidence-based curricula targeted at specific patient populations [75, 77, 78]; simulation and role play to promote effective compassionate communication with patients and their families [74, 91, 97]; clinical instruction and community service [79]; vignettes focused on improving patient and family centered care [105]; reflective practices aimed at increasing learners’ subjective understanding of the patient experience, compassion and confidence in clinical work [80, 100, 106, 107]; and complementary therapies, including Healing Touch [73] and Compassionate Touch [64] as nonpharmacological approaches to patient care. Individual learners’ coping and resilience skills were addressed through rounds that were implemented to encourage learners to share their own experiences and insights, own their vulnerabilities and support each other [81, 98]; complementary therapies such as Healing Touch [73] and Reiki [65]; and contemplative therapies, including mindfulness and compassion-oriented practices, meditation, and mind-body therapies [66, 67, 69–72, 82, 92, 93, 101–103].

#### Reasoning

Factors associated with the resource that impacted the cognitive or emotional decisions and behaviors of the learners and enabled or hindered the success of compassion training for practicing healthcare providers included those relevant to teaching, learning, and the organization.

##### Teaching mechanisms

Teaching mechanisms were defined as factors that impacted the process of sharing knowledge and experience during compassion training. Teaching mechanisms included highly qualified program leaders and facilitators, leadership and team learning activities, continuous learning, staff empowerment, education within the clinical context, discussion, and a variety of instructional approaches designed to enable learners to acquire the requisite attitudes, knowledge, skills, and behaviors to improve compassion.

Highly qualified program leaders and facilitators were an unequivocal requirement for the implementation of successful compassion training. Workplace-based programs benefited from an implementation phase where program facilitators with strong motivational and interpersonal skills provided training through mentorship, role modeling, and feedback to develop leadership and team capacity and instill a caring culture into the healthcare environment [83, 84, 95, 96]. Compassion training aimed at individual learners was optimized by professionally trained program facilitators and instructors who supervised learners, were familiar with the local healthcare culture, were good at explaining content, generated interest in the topic of compassion, created a safe environment, and provided support and helpful feedback [67, 85–87, 100]. Senior managers as facilitators were considered a potential barrier to compassion training, as their presence made learners less willing to disclose their shortcomings and conveyed a sense that teaching was addressing a deficiency in individual healthcare providers [98].

Leadership and team learning activities promoted relational ways of working and supported team members in providing compassionate care. Examples included developing a community of practice where managers and senior and junior healthcare providers worked together, implementing collective action plans, and conducting local team climate analyses, values clarification, team study days, and team discussions [18, 71, 73, 83–89, 94–96]. Some team activities also had negative effects, as learners felt ‘under examination’ or were reluctant to provide feedback to their coworkers [88, 94].

Continuous learning and sharing applied learnings with coworkers were necessary to sustain the learnings from compassion training. Tools and strategies that promoted continuous learning and actions included having clinical work covered by float healthcare providers to allow learners to consistently participate in compassion training, offering booster sessions after the initial intervention, and sending weekly reminders and tips about compassionate care for self and others [67, 70, 71]. Some learners maintained benefit from compassion training as they were motivated to make deliberate choices and pursue additional learning [65, 70, 101, 103]. In other situations, patient care demands and staff resourcing meant learners could not access consecutive training sessions, there was a lack of follow-up, or compassion training was too short to elicit change [64, 70, 78, 82–84, 88, 90, 92–95, 98, 99]; this prevented learners deriving full benefit from compassion training and embedding the learnings into routine practice.

Staff empowerment enabled learners to see themselves as innovators of compassionate actions. Compassion training motivated learners to explore issues within their practice and interpersonal relationships, to directly share ideas with their senior managers on improving compassion in practice, and to implement changes in their relational capacity [18, 66, 71, 83–85, 87–89, 95, 96, 99, 103]. Not all ideas were applied due to uncertainty about whose responsibility it was to authorize them, which was demoralizing for some learners [83, 84, 95].

Education within the clinical context guided learners to develop relational skills when interacting with co-workers and patients in their organization. Co-workers were able to pragmatically learn and face challenges together, often in the context of a busy clinical setting [18, 79, 83–88, 90, 95, 96, 98, 99, 103]. This approach required a social and emotional perspective rather than a focus on technical aspects of care, but some learners experienced anxiety about interacting with their co-workers and patients at such a personal level [88, 98].

Group discussion during compassion training included briefing and debriefing with program facilitators and other learners about patient-centered care and the impact of connecting and working with patients. Discussions worked well when ‘best practices’ were followed, which included defining the purpose of the discussion, ensuring the discussion addressed the learning objectives, encouraging learners to associate their own experiences with the events discussed, encouraging learners to actively participate and not critique others, and summarizing key learning points [94].

Several specific instructional approaches impacted compassion training. Contemplative practices were valued for their person-centered nature and the importance they placed on personal development [66–68, 70–72, 82, 92, 93, 101–103], but some learners lacked confidence in the practices, or experienced the practices as unnatural and embarrassing [93]. Didactic lectures and case-based scenarios were the primary components of evidence-based curricula [75–78]. Role play, simulation, video/audio recording followed by review, and patient storytelling provided safe opportunities for decision making in a realistic environment, and promoted reflection on patients’ lives and illness experiences [74, 77, 91, 94, 97, 100, 105]. However, role play and simulation only accommodated small groups, and some educators and learners were uncomfortable with these approaches [74, 91, 94].

##### Learning mechanisms

Learning mechanisms were defined as factors that impacted learners’ abilities to assimilate new knowledge, behaviours and skills during compassion training. Learning mechanisms included group participation, immediate feedback, recognizing and sharing positive practices, reflection, discussion, experiential/hands-on learning, mentoring, critical thinking and problem solving, and linking theory to practice.

Group sessions in a warm, welcoming, respectful and nonjudgmental atmosphere created a sense of belonging and a common goal, fostered support among learners, allowed learners to be models for each other in using the tools and practices provided by the training, and created networks in which learners developed critical friendships with peers, especially when the atmosphere was non-hierarchical [18, 73, 85, 87–89, 94]. For learners who felt intimidated by larger groups, a team approach or implementing a series of smaller group sessions was preferred [98].

Immediate feedback to learners from researchers, peers, patients or their families developed learning in a deliberate way. Feedback generated discussion on why particular interactions were effective and helped learners feel that their actions were legitimized. Learners became sensitized to the need to provide feedback to coworkers and commend them for their actions, even if the actions were part of the coworker’s job [18, 70, 83–86, 88, 89, 94–96]. Learners had to be accepting and receptive to positive and negative feedback and some learners needed time to reflect and plan before making changes in response to feedback [83, 84, 95, 96].

Recognizing and sharing positive practices with coworkers was a key component of workplace-based programs, where the focus was on what was effective. Statements were made about practices that worked well, and these statements were debated, defended and shared. Learners were committed to recognizing what works well, explaining why, and reframing their language to the positive. Learners found it refreshing to focus on positive development, felt a sense of hope and value, and adopted a new way of working with their coworkers and patients [86, 88, 89].

Reflection during protected time through the use of various media such as literature, art, writing and shared narratives enabled emotional resonance and a shared understanding of compassion to develop. Some learners considered reflection in action and reflection on action with critical analysis, a necessary part of their work and its inherent complexities. These learners valued the opportunities to build the skills of observation, interpretation and self-awareness as part of their professional development [81, 82, 98, 106, 107]. Other learners deemed reflective approaches ‘fluffy’, and preferred compassion training with a solid academic and scientific foundation conducted in a clinical setting [66, 97, 98].

Experiential/hands-on learning through role play and case-base scenarios allowed learners to explore roles that were different from their own, and provided learners the opportunity to reflect and make clinical judgments in a safe environment, form new concepts, and learn [64, 85–87, 91, 94, 97]. Experiential/hands-on learning enabled many learners to become aware of their own values and draw parallels with real life; however, some learners did not see the significance of the approach and expressed resentment at being required to participate in what they considered to be an uncomfortable experience [64, 91].

Other learning mechanisms associated with compassion training included mentoring, which provided a safe teaching environment where learners could reflect on real time situations and gain confidence in providing compassionate care [73, 99]; critical thinking and problem-solving, which were more helpful than memorizing and recalling facts [75, 77, 78, 85–87, 91]; and the ability to link theory to practice, which was appreciated as long as program content was not ‘theory heavy’, as some learners found this approach to be counter-productive and too much to process [85–87, 93].

##### Organizational mechanisms

Organizational mechanisms were defined as factors within the organization that impacted the process of sharing knowledge and experience with learners and learners’ abilities to assimilate new knowledge, behaviours and skills during compassion training. Organizational mechanisms included aligning activities with the priorities of the wider organization, time efficiency, flexibility in programming, and cost to the organization and the learner.

Implementation of compassion training was reliant on the extent to which it was valued by the wider organization and supported by senior staff. Institutional culture change and readiness were required to engage learners, generate acceptance and enthusiasm, and facilitate the implementation and sustainability of focused compassion training across organizations [18, 71, 79, 85, 88, 89, 93]. Compassion training programs were only feasible if the activities aligned with institutional priorities, the purpose and potential of the program was clearly communicated to staff by managers, processes were embedded into routine practice, leaders supported staff in their endeavors, roles were clearly defined, and adequate provision was made to introduce temporary or newly arrived staff into the program [64, 68, 83, 84, 86, 93, 95, 96, 98, 99].

Time-efficiency was a key component for learners participating in compassion training. Learners required protected time to participate in compassion training and implement their learnings into patient care. Managers’ wholehearted support was essential in allowing learners time to participate in the training. Team leaders needed to manage workloads to enable staff to attend compassion training [64, 92, 98, 99]. Some training was offered in an abbreviated format with dedicated online resources, as longer duration training was considered detrimental to enrollment [66, 67, 102, 103]. Other training had flexible scheduling to accommodate staff needs whereby learners were allocated to the training periods of their choice to make participation more convenient [78, 101].

The financial cost of compassion training to the organization needed to be balanced against the institutional benefits and cost savings associated with reduced staff absenteeism and improvements in the quality of care [68, 71]. In some cases, requisite time commitments and cost precluded compassion training from successfully influencing care [92, 94, 106]. The financial cost of compassion training also impacted individual learners. For some learners, compassion training was free of charge or offered for a nominal fee [69, 104]. Some learners were paid to participate in compassion training, a certificate of completion or a continuing medical education credit was issued, or learners were offered a prize [68, 70, 75, 102, 104, 105]. One program was considered a regular part of continuing professional development and was accredited by professional bodies [101].

### Outcomes

Outcomes of compassion training included learner experiences of compassion training; healthcare provider outcomes, including improvement in learner attitudes, knowledge, skills and behaviors; and clinical outcomes, including changes to the clinical process and benefits to patients. The majority of outcomes were qualitative or anecdotal, with little quantitative data substantiating outcomes. Learner self-report measures were used to evaluate changes in learner attitudes, knowledge, skills, and behaviors, as well as learner perceived benefits to patients. Very few records used patient or researcher report measures to evaluate outcomes.

Most learners reported that they enjoyed compassion training [85–87, 94, 97, 102], agreed that the training achieved stated objectives [68, 90], were satisfied with the facilitation, teaching, and workload of the program [64, 85–87, 103], and would recommend the training to their coworkers [68, 103]. Some learners reported a lack of confidence in the methods of teaching and the practices shared during training [64, 93, 94]. Other learners were concerned that practices would not be sustainable following training unless they were provided organizational support [70, 82–84, 95, 96].

Compassion training improved learner attitudes as they reported enhanced self-awareness and personal growth [65, 70, 73, 81, 101, 103]. Leaders participating in workplace-based programs reported an increase in their ability to lead the delivery of compassionate care, support peer learning, manage conflict, demonstrate consideration and empathy in interactions with patients, and to build trust with patients and their family [69, 72, 86, 87, 99]. Other learners perceived themselves as being more present and kind towards self, their co-workers, and patients and their families [65, 73, 93, 103, 106, 107]. Learners reported an improved sense of responsibility, accountability and professionalism, a more holistic view of patient context, and an understanding of the necessity to adapt their practice according to different patient needs [18, 75, 78, 88, 89, 106]. Learners were able to recognize areas for improvement and were empowered to instigate change [18, 88, 89]. However, some were unable to optimize work routines despite incorporating practices learned during compassion training into their work day, although the reasons for this were not clear [103]. Compassion training had a favorable impact on learners’ perceived stress, self-efficacy, self-compassion, and criticism of others, including coworkers and patients [65–67, 92, 103]. Learners were better able to work as a team, learn from each other, and support each other’s wellbeing [18, 88, 89]. Learners reported being less judgmental of others and were more confident in asking questions of patients and their families [18, 88, 89]. Learners were reminded to step beyond the biomedical aspects of care and be more mindful of the spiritual aspects of life, and to better process work experiences so they could build an action plan for managing challenging situations. This led to increased sensitivity towards others’ perspectives and fewer assumptions about how patients might wish to be cared for [65, 70, 76]. Learners reported reduced burnout, emotional exhaustion, anxiety and perceived stress, an improved emotional balance at work, an increased feeling of personal accomplishment, less frustration and anger, and a renewed sense of enjoyment of work [66, 70, 103].

Compassion training improved learner knowledge, skills and behaviors, as learners applied what they had learned during compassion training within the workplace, including communication and listening skills, clinical knowledge, symptom management, and exemplifying compassion and respect in their practice [75–77, 90, 94, 97, 105]. Learners were more confident in providing compassionate care using nonpharmacologic therapies, which were adjunctive to medications in addressing patients’ needs [64, 73]. Learners gained new insights into existing routines, which often signposted deficiencies in the care they currently provided [18, 88, 89]. Learners reported applying their training for self-care and to train their coworkers [69, 73]. Learners’ interest in personal self-care was sustained as they implemented an ongoing personal diet and fitness routine and pursued further education in healing arts [67, 103].

Compassion training reportedly impacted the workplace and patient care. Training fostered workplace cultures that were more reflective, open to learning, mutually supportive, and innovative [18, 83–89, 94–96, 99]. There was a recognition that each healthcare provider, whatever their role, had expertise that could be shared for the benefit of other team members [18, 88, 89]. Creating protected spaces in which team members could ‘take shelter’ promoted valued learning from each other, the opportunities for joint problem-ownership and a shared pursuit of solutions, and support in addressing patients’ needs [83, 84, 95, 96]. Relationships within healthcare teams were also reportedly improved [83, 84, 95, 96]. Some learners were able to bring calm to their coworkers and the working environment, and enhance team morale, such that healthcare providers became more emotionally connected, gave each other positive feedback, and celebrated good practice in their efforts to improve as compassionate practitioners [18, 65, 70, 73, 88–90]. Learners were more confident in approaching conflict and worked together to create new initiatives for improving human interactions, such as a poster showing the person-centered language they valued [18, 88, 89]. Learners noted that all staff needed to be trained to sustain compassion in the workplace as the absence of whole staff training could create a sense of isolation and dissonance among non-trained staff [78, 93].

Compassion training was also reported to benefit patients with learners reporting increased knowledge about, and comfort in, providing compassionate person-centered care and prioritizing conscious and deliberate engagement with patients and not simply confining their care to clinical tasks [18, 88, 89]. Learners’ assumptions about patients and the perspective of family members were challenged and their perceived ability to build trust with patients and their relatives was increased, which led to changes in how learners communicated with patients and their families [88]. Researcher-rated and patient-reported observations of emotional care and learner-reports of empathy suggested a reduction in negative interactions between staff and patients and fewer patient experiences of poor emotional connection with staff [95, 96]. Some learners reported that their practice was already compassionate; with compassion training providing an opportunity to further fortify their commitment to providing compassionate care [67, 103]. Learners reported being better able to help patients and families understand patient distress, which caused a change in patient thinking and behavior, with a reduction in patient self-criticism [93]. Learners reported that adopting a holistic approach to patient care improved pain management and enhanced healing and patient well-being [73]. Some learners reported decreased unplanned work absences following compassion training [69] and one multistep, multiprofessional, multiyear program that incorporated cultural transformation of the institution and healthcare provider training improved patient satisfaction, decreased patient safety events, and improved the national status of the host institution [71]. Compassion training benefits were often not sustained. The need for longitudinal learner development and a shift from a strict task-driven approach to a compassionate care approach often required a longer duration of training and organizational support [64, 70, 78, 82–84, 88, 90, 92–94, 98, 99].

## Discussion

To our knowledge, this is the first realist synthesis that identifies the contexts and mechanisms that are commonly associated with the outcomes of compassion training and conceptualizes the relationships between these factors through a program theory (Fig. 2). The results of this research are intended to inform the design and implementation of a compassion training program that is relevant to practicing healthcare providers in various settings, and that is accessible, sustainable, and supported over time (Table 3).

**Table 3:** Recommendations for the development of a compassion training program

• Senior leadership training module is a part of the training program • The program honours and aims to enhance healthcare providers’ innate compassion in order to avoid misconceptions that training is intended to address deficiencies on the part of learners • A learners’ needs assessment is conducted prior to training • Program content maps to the dimensions of compassion • Program incorporates a mix of experiential, online and in-person teaching • Facilitators embody compassion including a co-facilitator grounded in clinical practice and the organizational culture • There is a commitment to sustaining compassion and providing learners with educational resources after the program • The program is evaluated with valid and reliable measures, including a compassion measure	

The need to develop, enhance and sustain a culture of compassion in complex healthcare systems is well recognized [30, 46–51]. Current healthcare cultures are strongly driven by productivity and efficiency, which increases the numbers of patients treated and decreases healthcare costs but has an inadvertent negative impact both on staff morale and well-being, and on compassionate patient care [108]. One of the strongest findings from the current review was the requirement for organizational leadership and support when offering and sustaining compassion training for healthcare providers. Consistent with this, there has been a call for ‘compassionate design’, whereby organizations optimize compassionate engagement between staff and patients through the design of their services and by demonstrating their commitment to the values and behaviors that support compassion—literally and metaphorically embedding compassion in their bricks and mortar [108]. Other indicators of organizational commitment to the delivery of quality compassionate care include a well-articulated vision statement of values and philosophy, adequate structure and physical environment, and an innovative, facilitative, encouraging and empowering approach to management [109].

Organizations must nurture their capacity for compassionate care by involving senior leaders, who have a symbolic and practical role in creating and sustaining a patient-centered approach to healthcare [4, 86]. The present review indicated that training senior leaders had a ripple effect that sustained compassion training programs over time, had a positive impact on healthcare providers, and improved the overall patient experience [87, 110]. Compassionate senior leaders were important mechanisms that enhanced compassion training in individual healthcare providers. Offering compassion training in an organizational culture that was not supportive, did not emulate compassion, foster a safe learning environment or nurture a clinical culture of compassion, was a futile endeavor [86]. Despite these findings and the evidence that shows systemic failures in healthcare occur when leadership is lacking [30], most of the compassion training programs identified in this realist review targeted frontline healthcare providers and not senior leaders.

Teaching methods associated with successful compassion training adhered to current education theory and practice, and recommendations for professional development programs, such as embedding curricula in an empirical framework, adopting workplace learning and ensuring transfer of learning to practice, implementing programs that extend over time, fostering community development, and establishing support within the larger organizational context [111]. A previous systematic review described a diverse array of compassion training programs in healthcare [52]. These included leadership and team compassion training based mainly in the workplace and a variety of participatory programs for individual healthcare providers. The programs were feasible to implement and learners reported benefits to personal well-being, self-care, and perceived improvements in the quality of their interactions with patients [52]. This realist review demonstrated that the most effective compassion training programs for practicing healthcare providers involved contemplative practices, group discussion, and experiential learning conducted in a psychologically safe group setting with highly qualified program leaders and facilitators who had knowledge of the clinical context within which the learners practiced [85]. In particular, tools and strategies that promoted continuous learning and action were an essential component of compassion training. It was necessary to find a balance between time to cover course content and the clinical demands and time constraints of the learners [83, 84, 88, 92, 93, 95, 96, 99]. Some programs offered compassion training in an abbreviated format [66, 67, 72, 78, 101–103]; however, there seemed to be a delicate balance, as compassion training could eventually became too short to elicit change [90, 99]. Lack of continuing education following compassion training was counterproductive and limited potential benefits [78, 82].

Engaging individual healthcare providers, the target learners of the majority of compassion training programs, presented a further dynamic affecting the success and sustainability of training programs. An inherent tension within these programs was the need to make participation voluntary in order to ensure learners were motivated rather than being mandated to learn. Ideally, all healthcare providers should participate in training, including those individuals who were not naturally inclined or did not have an affinity to the topic—individuals who could perhaps benefit the most from training. Conversely, while an affirmative inquiry and staff empowerment approach that recognized healthcare providers’ inherent capacity for compassion was important [18, 88, 89], those who strongly self-identified as compassionate presented another challenge, as they often felt that they were already sufficiently competent in compassionate care [67, 103]. The present review suggests that training the entire healthcare team yields the greatest return on investment, while recognizing that a mandated approach to compassion training can be counter-productive [18, 78, 83, 84, 88, 89, 95, 96]. This tension highlights the importance of engaging learners prior to the training intervention to allow for misconceptions to be mitigated, to honor their innate desire for compassion and the clinical challenges that impede it, to demonstrate the personal benefits of training, to understand individual learning needs, and to convey the message that the training program is intended to advance their compassion and not as remediation [71].

Ultimately, compassion training should be tailored and dosed to the specific needs of the organization, the needs of the learners, and the time constraints inherent in the clinical setting. This may be achieved by engaging the organization and healthcare providers proactively at the outset of training, involving senior leaders in pre-orientation sessions, conducting learner needs assessments, refining course content, and offering asynchronous learning opportunities. Program content should also map to the dimensions of compassion [11, 53] as the majority of programs identified in the present review were based on the philosophy and theory of existing leadership training programs, quality improvement, patient centeredness or teambuilding rather than compassion itself - which often functioned as a by-product or a secondary outcome of the training.

Organizational pressures such as financial instability or service reorganization that resulted in a lack of support for staff training, a lack of emphasis on staff wellbeing, and an eroded sense of urgency and purpose for compassionate care [83, 84, 90, 93, 95, 96, 98, 99, 102] were barriers that must be overcome to ensure that compassion training is impactful and sustainable.

The literature on compassion training in practicing healthcare providers is heterogeneous. The present review is the first to use realist methods to examine the context and the mechanisms underlying compassion training that facilitate change, regardless of healthcare setting, practitioner type, or intervention. Two previous studies have used realist evaluation (as opposed to realist synthesis) to explore context, mechanisms and outcomes associated with specific interventions designed to enhance compassion in healthcare. One realist evaluation analyzed three community and mental health case studies of Schwartz Rounds implementation. Data collection was through researcher observations, interviews with rounds attendees, and evaluation sheets circulated by the organization. Findings showed that staff valued Schwartz Rounds as a protected space for shared reflection and that strong leadership was crucial for implementation [98]. The other realist evaluation analyzed the ‘Leadership in Compassionate Care Program’ (LCC Program) to identify organizational factors that influence the development and sustainability of a culture of compassionate care. Data collection was through semi-structured interviews and focus groups involving staff, leaders and, senior individuals in the clinical settings involved in the LCC Program. Four elements were identified, including focus on relational practices and practice development, leadership, and the establishment of a strategic vision and infrastructure [86].

The present review has several limitations. Relevant studies could have been missed due to the search strategy, underreporting of unsuccessful compassion training programs, and because included articles were restricted to the English language. The outcomes of compassion training are challenging to interpret and evaluate. Most studies used self-report to assess compassion-related outcomes, and few included external assessments by preceptors, peers, or most importantly patients. The majority of healthcare providers were nurses and the majority of were female, which is important because previous research shows that females may have higher levels of compassion for others than males [112, 113]. Finally, most studies were short-term; therefore, the long-term outcomes of compassion training on clinical processes and patient care could not be established.

These limitations should be considered in perspective, as this review extends the results from our recent systematic review [52] and aligns with the findings from a thematic analysis of surveys and one-on one qualitative interviews with leaders and educators of compassion training programs that aimed to determine the tangible and intangible factors that affect the operationalization of compassion training programs in the healthcare setting, and was conducted in parallel (Sinclair S, Harris D, Kondejewski J, Roze des Ordons AL, Jaggi P, Hack TF: Program leaders' and educators' perspectives on the factors impacting the implementation and sustainment of compassion training programs: a qualitative study, submitted). Together, these comprehensive contributions to the compassion literature will inform the development of compassion training programs in healthcare organizations.

## Conclusions

In conclusion, this realist review identified that compassion training may engender compassionate healthcare practice if it becomes a key component of the infrastructure and vision of healthcare organizations, improves leadership at all levels, and betters the performance of individual healthcare providers. Multimodal compassion training that includes workplace-based learning, engages institutional participation, and uses valid measures to assess outcomes is most likely to equip practicing healthcare providers with the requisite attitudes, knowledge, skills, and behaviours to deliver compassionate care that promotes healthcare provider workplace wellness, benefits patients and their families, and creates a sustained culture of compassion in healthcare.

## Supplementary Information

**Additional file 1.** Search strategy.

**Additional file 2.** Data extraction form.

**Additional file 3.** Summary of compassion training.

**Additional file 4.** Nurses: Context Mechanism Outcomes matrix by study.

**Additional file 5.** Summary of Contexts, Mechanisms, and Outcomes.

**Additional file 6.** Learners’ characteristics.

## Data Availability

The datasets used and/or analysed during the current study are available from the corresponding author on reasonable request.

## Abbreviations

CMOs: Context, mechanisms, and outcomes
RAMESES: Realist and Meta-narrative Evidence Syntheses - Evolving Standards
LCC Program: Leadership in Compassionate Care Program’
