# Supplementary figures and images for: What works for whom in compassion training programs offered to practicing healthcare providers: a realist review
Source: BMC Med Educ. 2021 Aug 28;21:455. doi: 10.1186/s12909-021-02863-w (PMC8403363; doi:10.1186/s12909-021-02863-w)

## Additional file 1: Search strategy

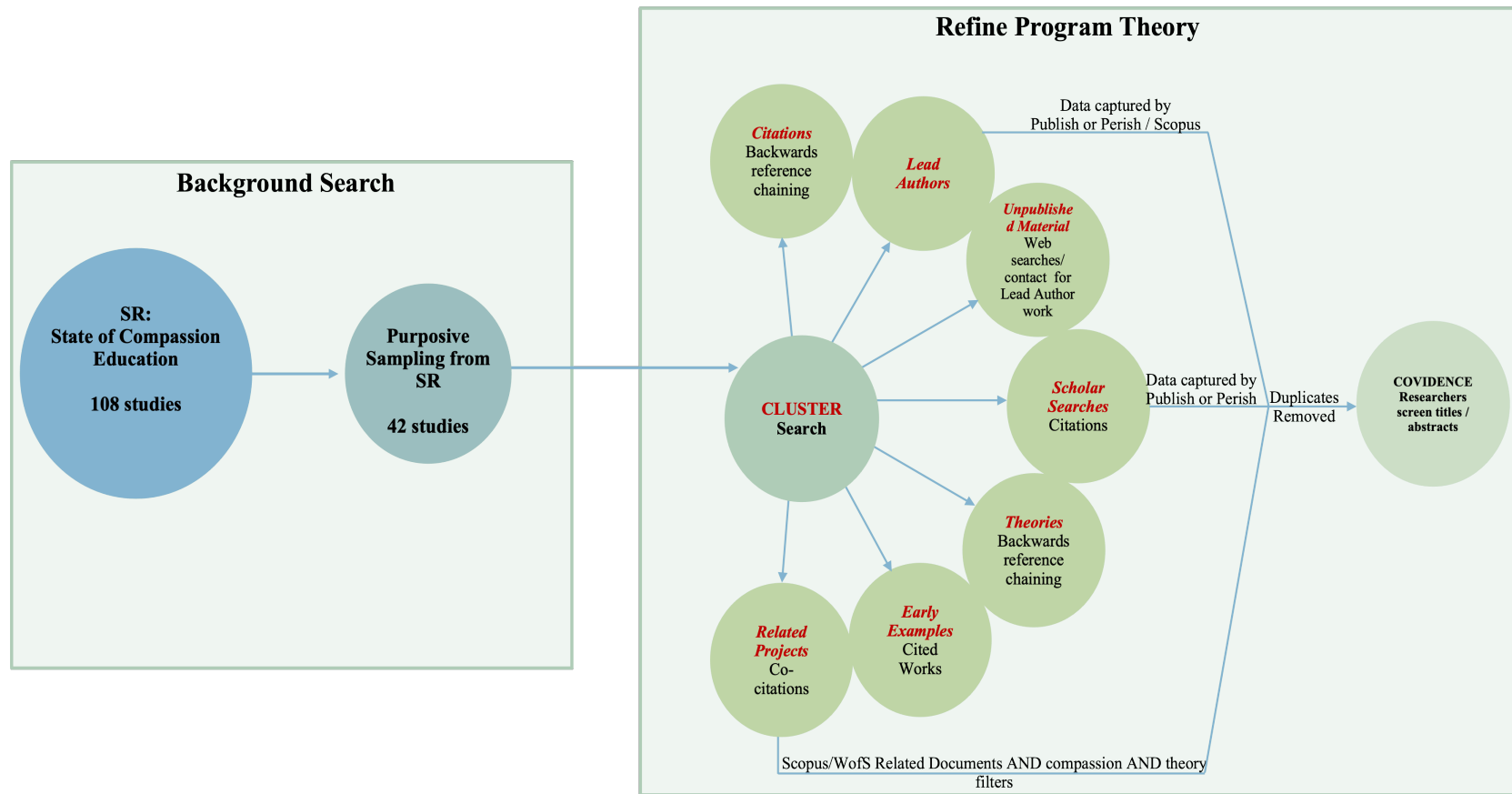

Supplement: Supplementary file 1 — Additional file 1. Search strategy. [file 12909_2021_2863_MOESM1_ESM.pdf]
