# Supplementary material for: What works for whom in compassion training programs offered to practicing healthcare providers: a realist review
Source: BMC Med Educ. 2021 Aug 28;21:455. doi: 10.1186/s12909-021-02863-w (PMC8403363; doi:10.1186/s12909-021-02863-w)
Supplement: Supplementary file 2 — Additional file 2. Data extraction form. [file 12909_2021_2863_MOESM2_ESM.pdf]

EnACT Realist Review

* Required

Email address *

Your email

Reference (Author, Year) *

Your answer

Study Location (Country & Setting)

Your answer

Study Objective(s)

Your answer

Study Design

- ◯ Qualitative
- ◯ Quantitative
- ◯ Mixed
- ◯ Observational
- ◯ RCT
- ◯ Other:

Participants (Learner Type + Academic Discipline)

Your answer

Sample Size

Your answer

Sample Selection

- ☐ Volunteer
- ☐ Mandatory
- ☐ Paid Incentive
- ☐ Other:

Intervention Description (workshop, computer-based modules, etc.)

Your answer

Definition of the Construct of Interest - e.g. is "compassion" or "communication" defined?

Your answer

Duration & Frequency of Intervention - e.g. 8 weeks, 1x per week for 5 hours

Your answer

Development of Intervention - why course offered, involvement of SMEs, how was course material devised, past experience, etc.

Your answer

Topics covered - e.g. mindfulness, communication, self-compassion

Your answer

Methods of Teaching & Learning - e.g. lectures, presentations, etc.

Your answer

Outcomes - include the tool used to measure the intervention effect

Your answer

Methods of evaluating outcomes (we are interested who is evaluating outcomes)

- include details of eval in "other" section like reflection, journaling, semi-structured interviews, standardized patient etc.

- [ ] Self-Report
- [ ] External Assessment
- [ ] Other:

Context where implemented - e.g. an epilepsy monitoring unit

Your answer

Intervention

Your answer

Actor

Your answer

Mechanisms (data driven or theoretical) e.g. correlation between variables; intervention resulted in feeling more confident or motivated

Your answer

Impact of Intervention on Outcomes

Your answer

Citation Tracking - references to follow up on

Your answer

Key words for additional searches

Your answer

Enviro Scan - e.g. an institution we might be interested in following up with

Your answer

Comments

Your answer

Questions

Your answer

Submit

Never submit passwords through Google Forms.

This form was created outside of your domain. Report Abuse - Terms of Service - Privacy Policy

Google Forms
