# Supplementary material for: What works for whom in compassion training programs offered to practicing healthcare providers: a realist review
Source: BMC Med Educ. 2021 Aug 28;21:455. doi: 10.1186/s12909-021-02863-w (PMC8403363; doi:10.1186/s12909-021-02863-w)
Supplement: Supplementary file 3 — Additional file 3. Summary of compassion training. [file 12909_2021_2863_MOESM3_ESM.pdf]

Additional file 3: Summary of compassion training

Reference	Training	Participants	Requirements for participation	Duration and Frequency	Development of the program	Content	Teaching methods (how information was shared)	Learning methods (how information was assimilated by learners)
**NURSES**								
Workplace based programs								
Acute care setting								
Bridges et al., 2019, 2018, 2017; Bridges and Fuller, 2015.	The Creating Learning Environments for Compassionate Care (CLECC) program.	Nursing teams working in wards with high proportions of older patients.	Recruitment of nursing teams was through ward manager agreement; it was expected that the ward manager would remain in post for at least six months.	A 4-month implementation period.	CLECC was designed by Jackie Bridges and Alison Fuller as a workplace learning program to promote change at unit/ward/team level by the development of leadership and team relational practices. Designed to enhance the capacity of nursing team members to relate to older people and hence support the delivery of compassionate care. Focus is to make reforms to routine practice and organizational resources.	Creation of an expansive learning environment which supports work-based opportunities to encourage the development of relational capacity across the work team. Development and installation of sustainable manager and team relational practices including dialogue, reflective learning and mutual support.	Led by a senior practice development practitioner/nurse. Regular CLECC meetings between ward manager and matron; ward manager action learning sets (small, facilitated group sessions) including one focused on influencing senior managers; team learning activities; peer observations of practice; team study days; mid-shift five-minute cluster discussions and twice weekly reflective discussions. Teams also developed a learning plan to be shared with a senior hospital manager that included sustainability measures for practices that underpin the delivery of compassionate care.	Dialogue, action learning, reflective learning, mutual support and role modelling.
Dewar and Cook, 2014.	A relationship centered appreciative	Nurses from 24 inpatient areas across one acute	By invitation of interested and motivated	1 year.	The use of Caring Conversations to allow the	Participants were supported to explore	Communities of practice were supported to work together (as appreciative leaders) to take	Dialogue, action learning through problem solving;

Reference	Training	Participants	Requirements for participation	Duration and Frequency	Development of the program	Content	Teaching methods (how information was shared)	Learning methods (how information was assimilated by learners)
	Leadership Program.	hospital.	participants.		development of knowledge about a person (staff member, patient or family member). Aims to help people develop a relational approach which enables them to work together to shape the way things are done and thus provide care that feels compassionate to patients, staff and families.	relationships with self, patients and families, within staff teams and the wider organization using Caring Conversations. Development of relational knowledge by understanding more about who people were, what mattered to them and how they felt about their experiences.	forward developments to enhance relationships and compassionate care. Action learning sets made up of peer groups and a facilitator explored issues in their practice using the Caring Conversations framework.<br>Work-based activities focused on learning about others' experiences were supported. Learners were provided with a resource book that contained a range of tools to help develop relational knowledge.<br>Feedback, reflection and support allowed learners to identify compassionate practices that worked well, to consider how these could happen more often and to test out different ways of relating with staff, patients and families to enhance compassionate caring.	reflective and experiential learning.
Dewar and MacKay, 2010.	Appreciative Action research project.	Staff on a medical ward caring for older patients.	Staff on Beacon Wards -selected by senior leaders as they had already demonstrated ways of working that exemplify a compassionate caring approach with staff, patients and families.	Part of a 3-year action research program – The Leadership in Compassionate Care Program.	Aims to achieve better quality compassionate care through a systematic analysis of what works well. Focused on development of compassionate care in clinical in-patient areas.	A range of data generation activities were used to identify what worked well and what were key processes in the delivery of compassionate care.	Phase 1 – Discovery-What is working well? Participant reflection and feedback, photo elicitation.<br>Phase 2- Dream? – What would be the ideal working environment? Feedback sessions and group interviews to share beliefs and values.<br>Phase 3: Design – What do we have to do to achieve our ideal? Group discussions to generate provocative statements and	Dialogue, action learning, reflective learning.

Reference	Training	Participants	Requirements for participation	Duration and Frequency	Development of the program	Content	Teaching methods (how information was shared)	Learning methods (how information was assimilated by learners)
							action plans. Phase 4: Destiny – What has worked well and how can people be supported to develop further? Reflective feedback on a one-to-one basis and development of action plans.	
Saab et al., 2019.	The Leaders for Compassionate Care Program (LCCP).	Nurses or midwives predominantly in management roles with extensive clinical experience.	Participants were recruited directly through the Hospital Group Chief Directors of Nursing and Midwifery in Ireland.	A 3-day program, the final day took place 6-8 weeks after the first 2 days.	The LCCP was a development program for nursing and midwifery leaders facilitated by the Florence Nightingale Foundation in the UK and launched in Ireland in July 2015. Provides several opportunities for leaders to learn about patient-focused quality improvement and compassionate leadership. Aims to empower leaders while supporting their teams.	Day 1-exploration of what 'leading for compassionate care' meant to the leaders; to elicit responsibilities and challenges faced in everyday practice. Consideration of the concepts of presence and personal impact. Linking of leadership to compassionate care delivery. Day 2-introduction to quality improvement, provision of tools and techniques to improve patient care and an introduction to co-consulting to build leadership practice experience. Day 3- sharing of	Theory was linked to practice; group work was encouraged, and critical thinking was promoted. The program used problem-solving approaches.	Dialogue, action learning and experiential learning.

Reference	Training	Participants	Requirements for participation	Duration and Frequency	Development of the program	Content	Teaching methods (how information was shared)	Learning methods (how information was assimilated by learners)
						examples of changes implemented following the LCCP and the impact of LCCP on clinical practice.		
MacArthur et al., 2017.	A critical analysis of the Leadership in Compassionate Care Program (LCCP).	Subjects - those receiving the LCCP Practitioners -those translating the program theories into practice. Policy Makers – those influencing the direction of the program.	Invited to participate.	7-9 months.	The LCCP was a three-year initiative funded by a benefactor and conceived in 2007. Aimed to embed compassionate care as an integral aspect of all nursing practice and education. Developed in partnership between a health board in Scotland and a higher education institution.	The LCCP included a) Emotional touchpoints - eliciting stories based on an individual's emotional experience of a number of 'touchpoints' during their healthcare journey.<br>b) Beliefs and values clarification - facilitation of staff groups to develop a common shared purpose/vision and understand how these influence practice and culture.<br>c) Photo elicitation - using photographs to prompt staff and patients to discuss	Four senior nurses and a lead nurse worked alongside staff conducting action research and facilitating innovative practice development content.	Dialogue, action learning and experiential learning.

Reference	Training	Participants	Requirements for participation	Duration and Frequency	Development of the program	Content	Teaching methods (how information was shared)	Learning methods (how information was assimilated by learners)
						the meaning of compassionate care, with statements subsequently being displayed as ‘positive care practices’.		
Landers et al., 2020.	An exploration of nurses’ and midwives’ views of the LCCP from the perspective of participating nursing/midwifery leaders, directors of nursing/midwifery, chief directors of nursing/midwifery, and facilitators who delivered the program.	Participating nursing/midwifery leaders, directors of nursing/midwifery, chief directors of nursing/midwifery, and facilitators who delivered the program.	Invitation after recruitment by non-probability purposive sampling.	Three modules delivered over three days with a 6-8 week gap between day 2 and 3.	Developed to support nurses to deliver compassion care. Aimed at providing nursing and midwifery leaders with the opportunity to reflect on their practice using a compassionate care lens.	Day 1: Facilitators explored the concepts of ‘presence’, ‘compassionate care’ and the ‘impact on self’ (of delivering such care).<br>Day 2: Group activities, discussion of quality improvement, leadership, networking and presentation.<br>Day 3: Facilitators delivered a session on managing change and the impact of LCCP on clinical practice is evaluated.	This study aimed to explore nurses’ and midwives’ views of the LCCP from the perspective of participating nursing/midwifery leaders, directors and chief directors of nursing/midwifery and facilitators delivering the program by using semi-structured interviews.	Not applicable.
Care home setting								
Dewar and MacBride, 2017.	Caring Conversations.	Staff in a care home.	Informed consent was gained before and after any data	10 months – over the first 2 months the study aims, and the nature of	Strong evidence exists about the importance of human interaction	Appreciative Inquiry (AI) was used with staff, residents and	Information was shared via feedback from observations and discussions arising from 1) photo elicitation using image	Dialogue, reflective learning.

Reference	Training	Participants	Requirements for participation	Duration and Frequency	Development of the program	Content	Teaching methods (how information was shared)	Learning methods (how information was assimilated by learners)
			generating activity.	participation, were explained to all staff. Following this staff were invited to join a core group to lead the study with the researchers.	in developing relationships that promote dignity and compassion. The Caring Conversations framework is a framework for delivering compassionate care based on human interactions that was developed in the acute healthcare setting. This intervention used the approach of appreciative inquiry (AI) to develop Caring Conversations in the care home setting.	families to identify skilled human interaction that promotes dignity. This was then used to inform an educational intervention that could be tested, refined and used more widely. In AI, the process of the research acts as the intervention.	cards where learners selected an image that summed up the meaning of the word 'dignity' and how they felt when communication worked well. 2) using the positive inquiry tool which poses two affirmative questions: 'what is working well for you here?' and 'how can your experience be improved?' By building on the knowledge they had acquired of what works well, learners developed small 'tests of change' that enabled these good practices to happen more of the time.	
Compassion training supporting individual healthcare provider level								
Acute care setting								
Stecker and Stecker, 2012.	Educational program to improve nurses' assessment of patients on the Epilepsy Monitoring Unit (EMU).	Nurses working on the EMU.	No information.	Educational program took place over 4 sessions, lasting around 45 minutes each.	Evidence-based educational component was designed through evidence gleaned from two Clinical Practice Guidelines, one systematic review, five descriptive	Review of the protocols for care of patients on the EMU. Patient evaluation points. Necessity of providing a safe environment for EMU patients.	Multi-modal teaching strategies: didactic, PowerPoint presentation, group discussions, case-based scenarios.	Reinforcement of existing protocols; application of knowledge to prioritize and plan patient care; simulated decision-making exercises.

Reference	Training	Participants	Requirements for participation	Duration and Frequency	Development of the program	Content	Teaching methods (how information was shared)	Learning methods (how information was assimilated by learners)
					design studies and one expert review. It was constructed for use in the project using "Brenner's from novice to expert model of development".			
Anderson et al., 2016.	Healing Touch (HT) practice group sessions.	Registered Nurses who had completed at a minimum HT level one through Inova Health System.	Voluntary; no paid incentive; free of charge course.	Monthly.	Founded by Janet Mentgen; evolved into a certification program; endorsed by the American Holistic Nurses Association.	Healing Touch: an authentic, mindful practice.	Training in HT techniques and processes, including the basic HT sequence.	Practice group sessions.
Palliative care								
Brown and Halupa, 2015.	An educational intervention about the needs of HIV/AIDS patients and families receiving palliative care.	Critical care nurses providing palliative care to HIV/AIDS patients.	Volunteers who were registered nurses in critical care with at least 3 months of critical care experience. Informed consent required.	A 4-and-a-half-hour course held fortnightly over 5 weeks.	The educational intervention was designed to increase the competency of critical care nurses in providing palliative care to HIV/AIDS patients. Source of the intervention is not reported.	Overview of HIV/AIDS palliative care together with common health and physical assessment skills, pharmacological interventions, nutritional measures and communication skills. Emphasis on issues such as the stigma experienced by patients with HIV/AIDS.	The course included PowerPoint presentations, videos, handouts, and class participation.	Action learning. Use of new earning when providing care in daily practice.
Chan, 2018.	The Compassion And Respect at the	Registered Nurses.	Voluntary; no paid incentive;	A single 3-hour workshop.	Using a patient family donation,	An introduction to: palliative care	A workshop. Learners received two CARES Tool Booklets, a	Use of the new learning in daily

Reference	Training	Participants	Requirements for participation	Duration and Frequency	Development of the program	Content	Teaching methods (how information was shared)	Learning methods (how information was assimilated by learners)
	End-of life (CARE) Program.		active employees of the hospital; informed consent given.		several oncology nurses collaborated and proposed the CARE Program to enable nurses to deliver compassionate, dignified, personalized nursing care at end of life consistent with patient and family wishes. They also aimed to decrease stress, burn-out, and compassion fatigue among staff.	and hospice care, the focus of palliative care, the hospital palliative care team at the hospital, effective communication for difficult topics, the CARES (Comfort, Airway, Restlessness, Emotional Support, Self-care) Tool, and the Care Cart resources (e.g., voice recorders, rosary beads, printed materials, knitted items).	nurses' version and a friends and family' version.	practice.
Betcher, 2010.	The Elephant in the Room Project. An intervention to improve nurses' communication with palliative patients and their families.	Nurses working on inpatient units with palliative care patients and their families.	No information.	A single intervention.	Developed by Denise Betcher, a Nursing Quality Coordinator after observation of nurses at work and feedback from them after a Palliative Care Education Day. This identified a need for expanded education on communication with palliative care patients and for the teaching to	Discussion of communication techniques, role playing, and simulation. Subsequently simulated conversations between actors playing palliative care patients and family and the nurses. Four scenarios were developed, and pairs of nurses participated in	45-minute didactic lecture followed by simulated conversations. These were videoed. All participants plus facilitators (a hospital chaplain and nurses from a local hospice) participated in a debriefing.	Role playing promoting the practicing of improved communication techniques. Self-reflection regarding personal practice further supported by debriefing with facilitators.

Reference	Training	Participants	Requirements for participation	Duration and Frequency	Development of the program	Content	Teaching methods (how information was shared)	Learning methods (how information was assimilated by learners)
					include interactive methods.	simulated conversations.		
Mental health setting								
Bunyan et al., 2017.	Motivational Interviewing (MI).	Nurses working on mental health inpatient rehabilitation wards.	All staff working on three mental health rehabilitation wards.	2-day training course followed by a 6-week frequent supervision period (twice weekly supervision sessions) and a 6-month ongoing supervision period (monthly supervisions).	MI is an established approach to helping people make positive behavior changes, through directive, person-centered counseling within a collaborative relationship between clinician and recipient.	Use of specific reflective listening skills by the practitioner to validate the patient's views, and to elicit and strengthen 'change talk'. Avoidance by the practitioner of the 'righting reflex' (the urge to 'fix' things through persuasion or advice for example, that is counterproductive for change).	Team training sessions were followed by supervised practice.	Using the MI skills in daily practice and during supervised sessions.
McEwan et al., 2020.	Compassionate Mind Training (CMT).	Mental health nurses from three NHS teams (two in-patient teams and one Crisis Intervention team).	A purposive sample of mental health professionals working at an NHS Trust local to the trainer and researchers. Informed consent given.	A standardized 2-day CMT training, followed by a focus group of 30 to 40-minute duration at the participants' workplace.	The CMT framework was originated by Gilbert in 2005 and is based on an understanding of the function and usefulness of human emotions. CMT is an emotion-regulation intervention and for this reason hypothesized to be particularly	Training was delivered by Gilbert and entailed: defining compassion; psychoeducation, including the three-circle model (a model outlining our threat, drive and soothing systems), old and new brain competencies (and how these conflict), and the	Multimedia and participatory elements.	Reflective and experiential learning. Use in daily practice.

Reference	Training	Participants	Requirements for participation	Duration and Frequency	Development of the program	Content	Teaching methods (how information was shared)	Learning methods (how information was assimilated by learners)
					useful within high-stress work environments such as mental healthcare settings.	concept of multiple selves (how our angry, sad and anxious selves all respond to stress); and practices, such as soothing rhythm breathing and three flows of compassion imagery (imagining having compassion for oneself, giving compassion to others and receiving compassion from others).		
Oncology setting								
De Souza, 2014.	Family Sculpting.	Nurses.	Pre- and post-registration, no other information.	No information.	Developed as part of a post qualification course for cancer nurses from the model of sculpting established by a multi-professional team at the Countess Mountbatten hospice led by a leading social worker in the field, the late	Students take the roles of family members. They are facilitated to create a physical representation of their relationships by, in turn, arranging themselves and other family members' bodies into an observable sculpture in response to different scenes or	Each student is given a character and some information about their character that they share with the group and some information that they do not disclose but use to condition their responses. The characters, once in role, introduce themselves and a genogram is drawn to help clarify relationships. Two students are asked to be observers. After 5 or 6 sculptors have worked the facilitator freezes the scene and participants reflect on the positions they have been placed	Experiential and reflective learning through discussion.

Reference	Training	Participants	Requirements for participation	Duration and Frequency	Development of the program	Content	Teaching methods (how information was shared)	Learning methods (how information was assimilated by learners)
					Frances Sheldon. Over time lecturers have learnt the technique through progressive observation and developed their own styles.	triggers such as a cancer diagnosis.	in. The scenario then moves onto an episode later in the trajectory of the patient's illness. At the end of the final freeze participants debrief. At this point the undisclosed tensions participants have been carrying are revealed which provides insight into the different preferences which have been revealed during the exercise.	
Mixed settings								
Schneider et al., 2018.	A nursing-service-learning partnership with a community of internally displaced persons in Columbia	Nursing professors and nursing students.	Students completing required community health practicums.	20-400 hours in the community.	The lack of services for the health needs of this community inspired the initiation by nursing professors of a service-learning partnership between an undergraduate nursing program in Medellin and the community's sole health center. The partnership allows nursing students to complete required community health practicums for courses in adult health, health	Nursing in under-resourced settings. The social determinants of health, the community nursing role including autonomy of practice and continuity of care. The scope of nursing practice including the opportunity to work at population level and intervene through policy and advocacy.	Learners participate in community diagnostics, home visits and health education sessions. These are followed by group debriefing discussions.	In-service experiential learning. Reflective journaling.

Reference	Training	Participants	Requirements for participation	Duration and Frequency	Development of the program	Content	Teaching methods (how information was shared)	Learning methods (how information was assimilated by learners)
					promotion, sexual health, occupational health, and/or nursing management within this community.			
Hawthornthwaite et al., 2018.	Patient Storytelling.	Nurses.	Participants at a nursing orientation.	No information.	Narrative medicine is a clinical practice of developing more empathetic relationships between patients and medical clinicians. Stories told directly by patients and listened to in-person by healthcare providers for discussion is a reminder of the need for patient and family centered care.	A two-part workshop prepares storytellers for their role. Storytellers are given background materials with prompts so that they can attend the workshop with a draft story describing moments from care that illustrates patient and family centered care or the absence of it. Storytellers are assisted with structuring and organizing their story and receive public speaking tips for effective delivery. At the following workshop storytellers	The nurse audience members listen to the patient stories and reflect on the care received.	Reflective learning.

Reference	Training	Participants	Requirements for participation	Duration and Frequency	Development of the program	Content	Teaching methods (how information was shared)	Learning methods (how information was assimilated by learners)
						rehearse their stories and are given supportive feedback so that they are confident to deliver their story in public.		
Wikland Gustin and Wagner, 2013.	The development of a teaching-learning model entitled 'The butterfly effect of caring' to enable participants' understanding of self-compassion as a source of the provision of compassionate care.	Four clinical nursing teachers who tutor students during their clinical placements.	Voluntary.	Participants met on 4 occasions for a total of 12-hours.	Initiated as a result of the need to develop a teaching-learning model that develops student's ability to be compassionate towards self and others whilst learning caring theory.	Study and experience of Watson's Theory of Human Caring with a focus on the first five of Watson's *10 Caritas Processes.*	Teaching–learning in relation to the understanding of clinical phenomena within caring practice. Participants learned about themselves, each other and compassion, while at the same time reflecting on how these activities could be used with students.	Dialogue, experiential and reflective learning.
Brathovde, 2017.	Reiki energy therapy for self - care.	Nurses working in an academic medical center.	The nurses were educated to a minimum of baccalaureate degree level and employed by the medical center. A convenience sample was selected from those who had previously voluntarily attended a 1-hr educational presentation introducing Reiki energy therapy as a self-care	A single training day following a1-hour introductory session.	A previous pilot study by Angela Brathovde had demonstrated that nurses perceived a positive change in their caring behaviors for themselves and others after learning Reiki Level 1.	Reiki is a form of alternative therapy which emerged in Japan in the late 1800s and is said to involve the transfer of energy from the practitioner's palms to their patient. Nurses learn the practice of Reiki in nursing, the proper hand placements for a Reiki treatment and techniques for	Personal review of the content of a 1-hr introductory session Multimodal teaching strategies: didactic followed by practice (led by Reiki Masters) of proper hand placement. Completed with attunement where students are connected to the universal Reiki energy source.	Regular practice of Reiki as a self-care technique.

Reference	Training	Participants	Requirements for participation	Duration and Frequency	Development of the program	Content	Teaching methods (how information was shared)	Learning methods (how information was assimilated by learners)
			practice.			a Reiki self-treatment.		
Mahon et al., 2017.	Mindfulness meditation.	Nurses.	All registered nurses working in 3 university hospitals were invited to undertake the mindfulness meditation intervention and take part in the study following an advertisement. Nurses who had already attended a mindfulness course or who already meditated and/or practiced mindfulness were excluded.	2-hours a week for 6 weeks in one hospital and 8 weeks in the other two hospitals. The shorter course spent less time focusing on self-compassion.	Mindfulness based stress reduction (MBSR), first developed by Dr Jon Kabat-Zinn in 1979 has been shown to reduce stress and increase self-compassion.	A mindfulness meditation and self-compassion training course.	No information.	Experiential learning.
Richards et al., 2006.	The Eight Point Program of Easwaran (EPP).	Hospital based nurses.	Current patient contact. Participants were recruited through in-service talks, flyers, and word of mouth. Participants were eligible for continuing professional education credit through the education department of the	Program classes were of a 2-hour duration over 8 weeks.	The EPP was developed by the late Eknath Easwaran. It is a non-sectarian spiritually based stress reduction program, - emphasis is to retrain attention so that a participant's focus in daily life is clearer and more directed. Development was	The EPP was presented as toolkit to promote concentration and gain control over intrusive thoughts. Topics covered included meditation, repetition of a holy word or mantram, setting priorities and slowing down, concentrating, training the senses	Home assignments included readings from the two texts, memorization of an inspirational passage of choice, and practicing with specific program tools (for example, slowing down). Experiences, experiments and readings of the week were discussed in small groups in the following session, allowing for feedback. Within these discussions the participants functioned as models for each other in using the tools of the program. Each meeting closed with a group	Dialogue, experiential learning through the practice of meditation.

Reference	Training	Participants	Requirements for participation	Duration and Frequency	Development of the program	Content	Teaching methods (how information was shared)	Learning methods (how information was assimilated by learners)
			host hospital. After completing all assessments, participants received stipends of $100.		based on two questions:<br>1) Is the EPP compatible with the philosophical perspectives and practical applications of nurses?<br>2) Can participation in the EPP effect positive changes in stressful work lives of nurses and their provision of care?	by overcoming conditioned habits and learning to enjoy what is beneficial, putting others first by gaining freedom from selfishness and separateness, finding joy in helping others, gaining inspiration from others and from texts.	meditation that lasted 10 minutes in the first session and progressively increased to 30 minutes by the end of the course.	
**CLINICIANS**								
Primary care								
Karkabi et al., 2014.	The use of abstract paintings and narratives to foster reflective capacity.	Family Medicine physicians or physicians in training.	Attendance at a workshop, informed consent given.	Single workshop.	Developed to facilitate reflective capacity by combining the use of abstract art and writing reflective narratives.	Three abstract paintings were presented to the learners. After choosing one they let the painting drive their emotions during quiet contemplation. Learners then vocalized the emotions that emerged while observing the painting. They then wrote a personal narrative	Learners pair -shared their narratives. Each answered questions about their narrative to promote reflective thinking. These included:<br>1. What ambiguity or contradiction, if any, was reflected in the narrative?<br>2. Where does this narrative take us? What challenges emerged within the narrative?<br>3. How did the narrative contribute to meaning making?<br>4. How might you do things differently in the future?<br>5. What could be an action plan?	Accessing feelings through the use of art as a contribution to reflective learning. Dialogue.

Reference	Training	Participants	Requirements for participation	Duration and Frequency	Development of the program	Content	Teaching methods (how information was shared)	Learning methods (how information was assimilated by learners)
						describing a meaningful or challenging situation with a student or a resident.		
Verweij et al., 2016.	Mindfulness-based stress reduction (MBSR).	General Practitioners (GP) trainers.	GP trainers could choose to attend. They are expected to participate in Continuing Professional development (CPD). The MBSR training accounted for 50% of the CPD for the year.	Either 1) 8 weekly sessions each lasting 2.5-hours, and a 1-day silent retreat between the sixth and seventh session; or 2) two full training days followed by four additional weekly evening sessions of 2-hours and a 1-day silent retreat during the weekend. All participants were asked to practice at home for about 30–45 minutes a day.	Burnout is highly prevalent in GPs. Mindfulness can reduce stress and burnout symptoms and increase empathy, self-compassion, and wellbeing. MBSR training specifically for GPs was developed.	The MBSR training followed the program developed by Jon Kabat-Zinn; however, the sessions were focused on the issues faced by GPs. All the sessions started with a brief presentation and discussion of the session theme in the context of clinical practice and supervising trainees. Themes discussed included awareness of pleasant or unpleasant sensations, feelings, or thoughts; perceptual biases and filters; burnout; boundaries or conflict	By practicing the mindfulness skills participants learned to focus their attention on the present moment and to observe their own thoughts, feelings, and behavior in a non-judgmental way.	Experiential learning by practicing the meditation skills.

Reference	Training	Participants	Requirements for participation	Duration and Frequency	Development of the program	Content	Teaching methods (how information was shared)	Learning methods (how information was assimilated by learners)
						management; and self-care.		
Schroeder et al., 2016.	A brief mindfulness-based intervention – the Mindful Medicine Curriculum (MMC).	Primary care physicians recruited from the family medicine and internal medicine departments.	Recruitment emails were sent to all physicians in these two departments. Inclusion criteria were (a) employed as a primary care physician by Providence Medical Group (PMG), (b) working at least 30% time in direct patient care, (c) aged between 25 and 75 years, (d) willing to be randomized to the intervention or waitlist control group (e) no prior participation in the same mindfulness-based intervention offered at PMG. Informed consent was obtained.	MMC is a 13-hour weekend training program plus 2-hour follow up sessions scheduled at 2 and 4 weeks after the weekend.	Mindfulness based interventions (MBI) have demonstrated effectiveness in reducing burnout, depression, anxiety, and perceived stress, and increasing well-being, resilience, and compassion for others among clinical and nonclinical populations. Traditional MBI programs can be time-consuming. This study used a brief MBI as described by Fortney et al., 2013.	MMC is similar to the protocol of Fortney et al., 2013 and is a modified version of Mindfulness-based stress reduction training (MBSR), with added elements of compassion skills training and brief mindfulness techniques designed to be used at work. The MMC was relevant to the physicians' workplace and emphasized the ability to incorporate mindfulness and compassion into interpersonal relationships.	Participants practiced applying mindfulness to the core clinical skills of Speaking, Listening, and Observing (SLO) using "SLO conversation" exercises.	Experiential learning using less than 10 minutes of formal mediation daily.
Fortney et al., 2013.	Abbreviated Mindfulness Intervention.	Primary care clinicians (primary care medical doctor or doctor of	Working more than 50% of time in direct patient care, availability	18-hours divided into sessions, Friday evening 3-hours, Saturday	Mindfulness is a form of mental training with the goal of generating	An abbreviated version of the 8-week Mindfulness-	Activities included guided sitting and walking mindfulness practices. Participants were encouraged to report their	Daily practice of mindfulness and the use of mindfulness

Reference	Training	Participants	Requirements for participation	Duration and Frequency	Development of the program	Content	Teaching methods (how information was shared)	Learning methods (how information was assimilated by learners)
		osteopathy, nurse practitioner, or physician assistant).	to attend most or all sessions, willingness to do 10-20 minutes mindfulness practice daily. Agreement to complete an online survey 4 times. Free course. Recruitment methods included e-mail invitations, flyers, visits to departmental meetings and clinics, and word of mouth.	7-hours, Sunday 4-hours, followed by two 2-hour evening sessions. 10-20 minutes daily mindfulness practice encouraged.	a greater sense of emotional balance and well-being. It has been helpful in reducing burnout among primary care physicians. The full 8-week course has high drop-out levels, so an abbreviated course was developed to lessen time constraints whilst having a similar effect.	based stress reduction (MBSR) program developed by Jon Kabat-Zinn. Training in mindfulness practices (sitting, movement, speaking, listening, and compassion for self and others) and their application to practicing medicine and everyday life.	experiences in a shared group setting, including examples of "practice in your practice" an adaptive mindfulness approach, that reflected working with patients using mindful attitudes. Audio CDs were provided for practice (Fourteen Essential Practices by S. Salzburg).A mindfulness website was designed specifically for this study to assist clinicians to bring mindfulness practice into the examination room. (www.fammed.wisc.edu/mindfulness)	(supported by instruction from a website) when seeing patients. Group discussion and reflection.
Palliative care								
Arnold et al., 2016.	Visual narrative.	Palliative care physicians.	Physicians completing a one-year palliative medicine fellowship at a large hospice were required to produce personal reflections narratives at the end of their training. Informed consent given.	Annual.	Aim was to elucidate palliative physicians' core experiences with their patients' dying and death using visual analysis of personal narratives containing images.	Fellows were encouraged to express their thoughts and feelings about death and dying through the arts and humanities.	The use of self-analysis to produce a visual narrative 1) exploring and expressing one's humanism, 2) reflecting on core experiences with death and dying, and 3) exploring the intimacy of the physician-patient relationship.	Personal reflection during and after the creation of a visual narrative.
Mental health								

Reference	Training	Participants	Requirements for participation	Duration and Frequency	Development of the program	Content	Teaching methods (how information was shared)	Learning methods (how information was assimilated by learners)
Riches et al., 2019.	Auditory hallucinations simulation.	Trainee and qualified clinical psychologists.	Participants were recruited as part of a general population sample who were invited to attend an immersive art exhibition. Advertisement emails were sent to the university mailing list.	A single intervention.	Simulation training may provide mental health clinicians with a subjective experiential understanding and a level of immersion that is lacking in role play. Auditory hallucination simulation could increase clinical psychologists' understanding and compassion towards voice hearers and have a positive impact on their clinical training and practice.	An immersive art exhibition which included an auditory hallucinations simulation. A pre-recorded audio guide described the artworks. Audio was repeatedly overlaid by "voices" performed live by professional actors. The actors performed voice "characterizations" which had been developed in workshops with young people who hear voices These aimed to reflect the range of auditory hallucinations, from positive to negative.	Each participant had a unique personalized voice-hearing experience. Participants received a full debriefing with psychologists after participating in the simulation.	Immersive experiential learning.
**MULTIDISCIPLINARY**								
High-risk populations								
Chambliss et al., 1990.	A participative AIDS/HIV training method.	Nurses and housekeeping staff.	All eligible staff members from units with HIV patients were required to attend training if there	2 group sessions 2-weeks apart.	Staff had previously attended a well-received didactic presentation of the hospital's policy	A participative HIV/AIDS training program designed to deal with the staff needs for an	An interactive shared problem-solving strategy. Problems were identified and the group generated solutions. Where information was lacking facilitators (doctoral level	Action learning through problem solving. Dialogue and group discussion.

Reference	Training	Participants	Requirements for participation	Duration and Frequency	Development of the program	Content	Teaching methods (how information was shared)	Learning methods (how information was assimilated by learners)
			was adequate staffing at the time of training. Informed consent was required.		on HIV/AIDS, current information regarding modes of transmission and risk reduction methods. Admission of the first HIV/AIDS-involved patient, however, highlighted the need for further staff training.	expression of, and possible resolution of, their concerns regarding the treatment of HIV/AIDS patients.	psychologists) and participants shared responsibility for seeking further information. At a following meeting the additional information was shared and its relevance to the previously proposed solutions discussed. After this meeting learners received a summary of the group-generated issues and answers.	
Palliative care								
Moore et al., 2017.	A "Compassion Intervention" to improve end of life (EOL) care for patients with advanced dementia and their families.	Nursing home and external healthcare providers. Informed consent was given.	Two nursing homes were invited to participate.	6-months.	An interdisciplinary care leader (ICL) with a background in social care was employed to facilitate the intervention under supervision from clinicians with palliative and dementia experience.	Content was led by the needs identified by managers at the nursing homes in consultation with the ICL. Included managing distress during hoist transfers, understanding pain and behavorial symptoms, dementia and EOL care, discussing EOL care with families.	Training, support and feedback from the ICL.	Use of the learning in daily practice.
Orellana-Rios et al., 2017.	Mindfulness and compassion-oriented practices.	Staff members of a palliative care center.	By invitation. Informed consent given.	10-week program. Initial 2-hour session	Exercises from *Metta* and *Tong-len* meditation	Mindfulness and compassion-oriented practices:	Participants were instructed to apply the learned techniques during work and to meditate	Experiential learning. Use of the learning in

Reference	Training	Participants	Requirements for participation	Duration and Frequency	Development of the program	Content	Teaching methods (how information was shared)	Learning methods (how information was assimilated by learners)
				and 9 weekly practice days. On practice days, staff members had the opportunity to participate in brief meditation sessions and one-to-one-sessions.	practices were adapted to be taught in a secular fashion directly at the palliative care unit.	1) development of a mindful presence 2) cultivation of Loving-Kindness 3) the practice of *Tong-len* in difficult situations and 4) the integration of practices into daily work situations.	daily at home. They received a CD with guided meditation exercises for home practice. Posters reminding them to breathe or walk mindfully and posters containing *Metta*-sentences (reminders of the attitude of Loving-Kindness) were displayed in staff rooms.	daily practice.
Rao and Kemper, 2017.	Online training in specific meditation practices.	Healthcare providers.	Modules were offered free of charge to healthcare providers at the university and at a small fee to individuals outside the university community.	No information.	Meditation is focused on building compassion and other positive emotions. Online training is cost-effective and convenient for busy HCPs.	Three online meditation training modules: (a) Gratitude-focused Meditation, (b) Positive- or Sacred-Word-focused Meditation (c) Loving-kindness/Compassion-focused Meditation.	Modules contained descriptions of each meditation technique, discussions of available scientific evidence regarding risks and benefits of each approach, links to guided practices to encourage experiential learning, suggestions for incorporating each technique into clinical practice, and pre- and post-module self-reflection exercises.	Experiential learning and self-reflection.
Mental health setting								
Suyi et al., 2017.	Mindfulness training – an abbreviated Mindfulness-based stress reduction program (MBSR).	Healthcare providers employed in mental health - psychiatrists, doctors, allied health professionals, case managers pharmacists,	(1) working at the Institute of Mental Health, Singapore, (2) above the age of 18 years, and (3) able to attend at least 4 out of the 6 sessions of	A 6-week program delivered once a week for 2-hours. Participants were requested to practice 30 minutes of formal meditation daily.	The MBSR program developed by Jon Kabat-Zinn was used as a guide in the development of the mindfulness program. The length of the	(1) Welcome and introduction to practice, (2) perception and engaging with practice, (3) awareness of being stuck in one's life and how	Meditation practices such as body scan, breath meditation, kindness meditation, mindfulness of feelings/thoughts meditation, and mindful movement/yoga were taught. A discussion period where participants were invited to	Experiential learning and group discussion.

Reference	Training	Participants	Requirements for participation	Duration and Frequency	Development of the program	Content	Teaching methods (how information was shared)	Learning methods (how information was assimilated by learners)
		researchers, or nurses.	mindfulness training. Participants excluded if they reported a previous diagnosis of psychosis, severe depression, borderline personality disorder, high suicide risk, and/or alcohol/drug misuse or dependence at any point in their life. Small monetary incentive.		program was shortened as a longer program is associated with a higher attrition rate.	to get unstuck,<br>(4) reacting and responding to stress, exploring perceptions and thoughts,<br>(5) mindful communication in stressful situations,<br>(6) cultivating kindness toward self and others.	share stories about their mindfulness practice, or thoughts on the topics presented during the training. Recordings of guided meditation were given to participants for use during homework practice.	
Elderly care								
Ross et al., 2013.	Simulation training for improving the quality of care for older people.	Doctors, staff and senior nurses, healthcare assistants and allied health professionals.	Entire interprofessional ward teams were able to attend as a group as a result of the planned closure of their ward.	1-day human patient simulation (HPS) course.<br>1-day ward based simulation (WBS) course.	Inpatient care for older people requires specialist skills to deal with their complex needs. Simulation training provides an immersive, dynamic environment in which learners can participate actively and practice skills in a risk-free	HPS scenarios used mannikins and actors. These included a gastrointestinal bleed, delirium, *Clostridium difficile* infection, a busy ward, the hospital at night and clinical communication regarding withdrawing patient care. WBS	During the HPS course staff participated in at least one role play. Each scenario was followed by a facilitated debrief and included a focus on reflective clinical practice. The WBS used a mixture of role plays, exercises using part-task trainers and an ageing suit to simulate the experience of being older. The healthcare assistants, nurses and physiotherapy staff were allowed to play a significant role before requiring medical	Active experiential learning which directly translates to daily practice. Reflective learning.

Reference	Training	Participants	Requirements for participation	Duration and Frequency	Development of the program	Content	Teaching methods (how information was shared)	Learning methods (how information was assimilated by learners)
					environment.	exercises included caring for a physically disabled patient, a multidisciplinary meeting, feeding and nutrition, physically caring for the older person and caring for relatives.	input. Each exercise had an integrated debrief encouraging reflective practice and focus on non-technical skills.	
Farr and Barker, 2017.	Schwartz Rounds.	Open to all staff including non-clinicians.	Invitation.	No information.	Developed by the Schwartz Centre for Compassionate Care as a form of group support for HCPs.	Evidence based interdisciplinary discussions where HCPs can share experiences of social and emotional aspects of care in order to foster improvements in patients care. Schwartz Rounds may focus on themes made up of a number of cases or focus on one particular case.	Opens with a short presentation from a multi-professional panel, facilitators encourage presenters to go beyond the facts and focus on their emotions in relation to the story they are telling. Audience members then share reflections, but do not problem solve or offer advice.	Dialogue. Reflective learning that can be used in daily practice.
Gale et al., 2017.	Compassion Focused Therapy (CFT).	Clinical psychologists and trainees, a psychotherapist.	Participants were recruited from CFT supervision groups and through a compassion conference. Informed consent was given.	Participants had already undertaken a variety of training in CFT: all had completed a 3-day introductory workshop; 4 had	Personal practice of therapy techniques can impact empathy for the client, therapeutic understanding, therapist skills and self-	Personal practice of CFT. CFT uses a multi-modal approach developing the skills of compassionate attention, imagery, thinking	Personal practice of CFT which was supervised for some learners.	Experiential learning through personal practice. Reflective learning.

Reference	Training	Participants	Requirements for participation	Duration and Frequency	Development of the program	Content	Teaching methods (how information was shared)	Learning methods (how information was assimilated by learners)
				completed advanced training and 2 had taken part in a personal practice workshop. 7 participants received CFT-specific supervision.	awareness. CFT requires personal practice during training, and on-going personal practice of Compassionate Mind Training (CMT) exercises is encouraged.	and reasoning, behavior and emotions. CFT draws on a range of standard therapeutic interventions and a series of CMT exercises specifically designed to stimulate compassion.		
Mixed settings								
Altamirano-Bustamante, et al., 2013.	A clinical ethics course.	Mexican healthcare professionals with current active practices in several clinical medical areas.	Invitation to participate in a free online course; awarded a 60-hour Continuing Medical Education (CME) certificate on completion.	60-hours.	Developed in response to the need to promote a relationship between evidence-based and values-based medicine by determining values relevant to everyday medicine. The course was designed by a cross-functional group including medical doctors, teachers, anthropologists, sociologists, philosophers and bioethicists.	Five modules: the person and human dignity, medical ethics, healthcare professional/patient relationship, clinical ethics committees, and methodologies for ethical discernment. Additionally, the course reviewed guidelines for ethical decision-making.	Online course. Exploration of personal values. Problem-solving exercises regarding how to apply ethical concepts and theories to ethical dilemmas. Use of patient simulation in clinical vignettes, motivational videos and an online discussion forum.	Action Learning. Reflective learning and critical analysis of ethical dilemmas in clinical practice. Use of the learning in daily practice.
Reynolds et al.,	Experimentally	Medical and	Convenience	A single	Healthcare	Learners viewed a	After viewing the slideshow	Induced

Reference	Training	Participants	Requirements for participation	Duration and Frequency	Development of the program	Content	Teaching methods (how information was shared)	Learning methods (how information was assimilated by learners)
2019.	induced compassion.	nursing students and qualified healthcare professionals.	sampling via social media and medical networks was used to recruit medical students and qualified health care professionals aged 18+ who were fluent in English. Interested participants were sent study information and a link to the online survey. Participants were entered into a prize draw for an iPad mini. Informed consent was obtained.	intervention.	professionals can find it hard to engage with patients who present with unpleasant symptoms and/or are to blame for their own health problems. Research suggests that inducing compassion might be a way of maintaining professional engagement in such circumstances.	2-minute slideshow. This had been previously validated to induce compassion and included images of either humans (13 images) or an animal (1image of a puppy) in various situations depicting helplessness, vulnerability, and physical and emotional pain.	learners rated their current emotions and were presented with 4 gender matched clinical vignettes that depicted hypothetical patients who varied in terms of the degree the patient had a responsibility for aggravating their condition and the presence of symptoms that might be seen as disgusting.	compassion was shown to offset disengagement from patients with challenging presenting features.
Han and Kunik, 2017.	Compassionate Touch (CT) for people with dementia (PWD).	Staff in residential care settings.	Staff who had attended CT coach training.	A 2-hour training session.	The CT program was developed by Ms. Ann Catlin (a registered occupational therapist and licensed massage therapist) in the AGE-u-cate Training Institute as a way of relating to PWD, especially in residential care settings. The	The CT coach training covers: understanding about PWD and their behaviors as expressions of their unmet needs; skilled touch vs. other forms of touch used in dementia; evidence for therapeutic touch; verbal and non-verbal	Hands-on practice and video-recorded CT training materials and handouts.	Experiential learning by using the CT program in daily practice and from training others in the technique.

Reference	Training	Participants	Requirements for participation	Duration and Frequency	Development of the program	Content	Teaching methods (how information was shared)	Learning methods (how information was assimilated by learners)
					program was designed to be used for reducing behavioral symptoms of PWD and unnecessary use of anti-psychotic medication.	communication techniques to make a connection with PWD; how to initiate the CT session and get permission to touch; non-verbal positive and negative responses to touch; skilled touch protocol on hands, back, and feet, and focused touch techniques.		
Penson et al., 2010.	Schwartz Rounds.	Multidisciplinary including doctors, nurses, chaplains, social workers and other staff.	No information.	1-hour.	Schwartz Rounds were developed to provide a multidisciplinary forum in which caregivers discuss difficult emotional and social issues that arise when caring for patients. Before the Rounds there was no real opportunity in the regular workday to discuss these issues in depth.	A very brief clinical history of the patient, including relevant information about the family situation, patient's attitude, and relevant circumstances is presented. Each panelist then describes his or her own perspective, narrating their experience and the psychosocial issue that will be the topic of the day's discussion,	Usually opens with a case presentation. Panelists and other caregivers in the audience ask questions and exchange experiences, thoughts, and feelings in relation to the topic. A facilitator helps lead and focus the discussion and summarizes the significant points at the end of the Round.	Dialogue. Reflective learning. Rounds commonly stimulate small follow up discussions among colleagues. Rounds have led to specific changes in institutional practices or policies.

Reference	Training	Participants	Requirements for participation	Duration and Frequency	Development of the program	Content	Teaching methods (how information was shared)	Learning methods (how information was assimilated by learners)
						which follows as an open-group exercise.		
Kemper and Hill, 2017.	Training in three integrative therapies – guided imagery, acupressure and Reiki.	Faculty staff, or those in training at the academic medical center.	Fee charged.	Guided imagery – two 3-hour workshops separated by two weeks. Acupresssure – two 4-hour workshops separated by 2 weeks. Reiki – single day long workshop of 8 -hours.	Increases in efficacy and self-confidence in providing compassionate care have been shown following professional training in complementary therapies allowing provision of nondrug therapies.	Introductory training in guided imagery, acupressure and Reiki.	No information.	Experiential practice-based learning.
Kemper et al., 2017.	Training in Mind-Body Therapies.	University medical students, faculty and staff.	Elective online course. Free to health professionals and trainees at the university; others were charged a nominal fee.	Herbs and Dietary Supplements (HDS) course – up to 14-hours of online training. Mind-Body Skills Training for Resilience, Effectiveness and Mindfulness (MBST) – up to 12-hours of online training.	Online mind body training has positive short-term benefits, this study assessed the dose-response relationship between the number of hours of online mind-body skills training for health professionals and relevant outcomes a year later.	No information.	Online learning.	Experiential learning. Frequent practice of mind-body skills was associated with more benefit.
Moffatt-Bruce et al., 2019.	Mindfulness In Motion (MIM) training, "flipped classroom" mindfulness training, Gabbe	MIM for ICU personnel, "flipped classroom" mindfulness training for faculty	Free programs, voluntary participation.	MIM is an 8-week program of a 1-hour/week intervention. "Flipped classroom" is an	Developed at the organizational level to reduce burnout promote staff engagement and improve	MIM consists of gentle yoga, mindfulness training, music, nutrition and self-assessment with	Didactic learning and guided meditation practice. Weekly reminders of mindfulness tips sent out by email during the initial 8-week intervention, followed by weekly "Mindful	Experiential learning by practice of the techniques learnt and participation in wellness

Reference	Training	Participants	Requirements for participation	Duration and Frequency	Development of the program	Content	Teaching methods (how information was shared)	Learning methods (how information was assimilated by learners)
	Health and Wellness program	and residents, Gabbe Health and Wellness program for staff, faculty and residents across the entire medical center.		intervention phase consisting of two components of up to 7-hours of training. Participants are also strongly encouraged to attend three 1-hour in-person interactive lectures on mindfulness. The Gabbe Health and Wellness program comprises a comprehensive offering of the MIM programing, including weekly and monthly MIM boosters.	health care outcomes.	coworkers. "Flipped classroom" consists of 4 online modules on mind-body skills training: (1) introduction to stress, resilience, and the relaxation response, (2) guided imagery for pain, insomnia, and behavioral issues, (3) autogenic training, and (4) the relaxation response: clinical, cognitive, and emotional effects. It is followed by 3 interactive discussion sessions. The Gabbe Health and Well- ness program comprises MIM in addition to wellness walks, integration with the Ohio State University Wellness Series, yoga, Zumba sessions for all staff, and Gabbe	Moment" emails to all former participants.	activities.

Reference	Training	Participants	Requirements for participation	Duration and Frequency	Development of the program	Content	Teaching methods (how information was shared)	Learning methods (how information was assimilated by learners)
						Wellness retreats.		
