# Supplementary material for: What works for whom in compassion training programs offered to practicing healthcare providers: a realist review
Source: BMC Med Educ. 2021 Aug 28;21:455. doi: 10.1186/s12909-021-02863-w (PMC8403363; doi:10.1186/s12909-021-02863-w)
Supplement: Supplementary file 4 — Additional file 4. Nurses: Context Mechanism Outcomes matrix by study. [file 12909_2021_2863_MOESM4_ESM.pdf]

Additional file 4:

Nurses: Context Mechanism Outcomes matrix by study

Reference	Context	Mechanism		Outcomes (intended or measured)
		Resource	Reasoning	
**NURSES**				
Workplace based programs				
Acute care setting				
Bridges et al., 2019, 2018, 2017; Bridges and Fuller, 2015 (Medicine for older people and surgical wards).	The need to strengthen the delivery of compassionate care in UK health and social care services, in particular to older patients, has been consistently identified as a high priority by policymakers in recent years.<br><br>Training, staffing levels, leadership, motivation and organizational culture are all implicated in failures of care.<br><br>Leadership and team practices, such as	Team-based educational program entitled Creating Learning Environments for Compassionate Care (CLECC) focused on developing manager and team practices to create an expansive learning environment.<br><br>A "boost" version after the initial implementation period was used to refresh ideas and boost activities and principles.<br><br>An enhanced version included additional features with a focus	The program was introduced to the workplace during a 4-month implementation period.<br><br>The program promoted relational ways of working between nurses:<br><br>• Monthly ward leader action-learning sets.<br><br>• Team learning activities, including local team climate analysis and values clarification.<br><br>• Peer observations of practice and feedback to team by	**K1 Reaction:** Nurses valued the principles behind CLECC. Beyond the activities nurses were directly involved in, they struggled to visualize the purpose and potential of CLECC. The principles that underpin CLECC appeared to be well embedded into the teams, but the activities that support these principles had not continued on all the wards 12 months after the start of CLECC.<br><br>**K2a Attitudes**: Levels of empathy varied *. Nurses saw the CLECC as a way to build the team and improve care.<br><br>**K4b Benefits to patients:** The odds of a negative interaction were not significantly reduced because of the effect of the CLECC intervention†. Patient evaluations of emotional care were not significantly changed because of the effect of the CLECC intervention**.

Reference	Context	Mechanism		Outcomes (intended or measured)
		Resource	Reasoning	
	role modelling, mutual support, reflective learning and dialogue, required to support nursing staff in their caring role are unlikely to be in place in most care settings.	on improving managerial support.	volunteer team members.<br><br>• Team study days focused on team building and understanding patient experiences.<br><br>• Mid-shift 5-minute team cluster discussions.<br><br>• Twice-weekly team reflective discussions.<br><br>• Mutual support.<br><br>• Dialogue.<br><br>The CLECC property of plasticity enabled nurses to develop and adapt practices that suited local circumstances.<br><br>The CLECC empowered nurses and gave them the	

Reference	Context	Mechanism		Outcomes (intended or measured)
		Resource	Reasoning	
			opportunity to see themselves as innovators.<br><br>Lack of follow-through for ideas for improving practice on the ward was demoralizing for the nurses involved, who were keen to put forward ideas and action plans.<br><br>Confusion among individuals as to whose role it was to make CLECC happen.<br><br>Implementation was dependent on the extent to which the activities harmonized with the priorities of the wider organization and communication of the value of the program to frontline workers by managers.<br><br>CLECC activities were compromised when	

Reference	Context	Mechanism		Outcomes (intended or measured)
		Resource	Reasoning	
			patient care demands were very high and staffing resource was low.<br><br>The extent to which the ward team perceived that they were supported in their endeavors by the matron was viewed as a strong mediator of whether or not CLECC was a success in influencing care.<br><br>There was no provision for inducting temporary or newly arrived nurses into CLECC, limiting their opportunity to make sense of CLECC.<br><br>The program was feasible to implement and may be of benefit in acute care settings when the local conditions are conducive. When conditions are not	

Reference	Context	Mechanism		Outcomes (intended or measured)
		Resource	Reasoning	
			conducive, it may not be possible to implement or, if initially implemented, not possible to then sustain it.	
Dewar and Cook, 2014.	The pressure to develop more effective leadership practices resulting in meaningful improvement in patient care, and changes in culture, is increasing.	Appreciative inquiry (AI), which involves appreciating what was happening, reflecting this back to staff, and facilitating discussions and action to help them to develop their practice and make this more compassionate.	Senior staff valued the program.<br><br>Learning together in context.<br><br>Communities of practice (manager, charge nurse/ward manager, senior registered nurses and junior registered nurses) were supported to work together to take forward developments to enhance relationships and enhance compassionate care within their area.<br><br>Action learning sets made up of a band of specific peer groups with a facilitator.	**K2a Attitudes**: Nurses were more self-aware; nurses reported enhanced relationships with the team, patients and patients' families and being more sensitive to the perspective of others and more open and sharing of themselves. Nurses were motivated to learn about others' experiences and use this as a platform for continuous improvement. Nurses were committed to improving the care experience for patients, families and staff.<br><br>**K3 Behaviors:** Nurses reported using different conversations in the workplace with patients and to resolve difficulties in the workplace. Clinical nurse managers used the Caring Conversations framework to underpin their meetings with senior managers.

Reference	Context	Mechanism		Outcomes (intended or measured)
		Resource	Reasoning	
			Nurses were able to explore issues in their practice using the framework of Caring Conversations.<br><br>Work based activities to develop relational knowledge by understanding more about who people were, what mattered to them and how they felt about their experiences.<br><br>Reflective spaces.<br><br>Staff culture questionnaire prompted feedback and discussion.<br><br>Being part of the program created networks for people to use peers as supporters and critical friends.<br><br>Tools and strategies that promoted continuous learning,	

Reference	Context	Mechanism		Outcomes (intended or measured)
		Resource	Reasoning	
			reflecting and action rather than a set of defined management and leadership content.	
Dewar and Mackay, 2010.	There is increasing focus in policy, practice and research about the importance of the caring dimension in healthcare as well as an ever increasing strive towards meeting targets, increasing patient throughput and working within financial constraints.<br><br>There is also emphasis on strengthening the climate for care, promoting models of practice that are centered around relationships, and the need to nurture and sustain core fundamental person centered caring skills	AI, which involves appreciating what was happening, reflecting this back to staff, and facilitating discussions and action to help them to develop their practice and make this more compassionate.	Researchers spent time building relationships with nurses.<br><br>Feedback to nurses who enjoyed reading the excerpts from the data and stated that having them in print made them feel that their actions were legitimized.<br><br>Making statements about practices that worked well and generating positive care practices that could be shared debated and defended.<br><br>Matching statements to images and displaying these as a rolling program via a digital photoframe at the nurses' station.	**K3 Behaviors:** Actions were developed, implemented and evaluated to develop: 1) Knowing who I am and what matters to me, 2) Understanding how I feel, 3) Work with me to shape the ways things are done, which had relevance for nurses, patients and families. Nurses tried to get to know each other and some patients and families as people. Nurses engaged at an emotional level with patients and families.

Reference	Context	Mechanism		Outcomes (intended or measured)
		Resource	Reasoning	
	and values.		Discussion of the statements.<br><br>Development of key questions to learn more about patients as people.<br><br>Development of individual and collective action plans.<br><br>In the initial phase, nurses felt pressure to do well.<br><br>Nurses were emotional and anxious when interacting with patients.<br><br>Shift patterns and the busyness of the ward was a barrier to sharing with a wider team.	
Saab et al., 2019; MacArthur et al., 2017; Landers et al., 2020.	A lack of compassionate leadership has a negative impact on healthcare outcomes	Development program providing experiential and highly interactive learning with other	Organizational funding.<br><br>Knowledge and expertise of the program facilitators.	**K1 Reaction:** Nurse leaders enjoyed the program and were satisfied with the teaching, assessment, workload, organization and infrastructure.

Reference	Context	Mechanism		Outcomes (intended or measured)
		Resource	Reasoning	
	and quality, as highlighted in two key reports in the UK: *Report of the Morecambe Bay Investigation* (Kirkup, 2015) and the *Report of the Mid Staffordshire NHS Foundation Trust Inquiry* (Francis, 2013).<br><br>Within these reports, the failure of several nursing leaders in their role and responsibility to care was identified as one of the key contributors to detrimental, neglectful and systemic failures to safeguard a culture of safety, quality and compassion (McSherry and Pearce, 2016).	leaders from a wide range of services and specialties.	Nurse leaders were recruited.<br><br>Problem-solving approaches as opposed to rote recall or memorization of facts.<br><br>Program facilitators were good at explaining content and made the subject interesting.<br><br>Program facilitators provided support and helpful feedback.<br><br>Group work.<br><br>Critical thinking, reflection, and linking theory to practice.	**K2a Attitudes:** Nurse leaders felt confident to work as members of the multidisciplinary team, to demonstrate consideration and empathy in interactions with patients and to build trust with patients and their relatives and lead in compassionate care delivery.<br><br>**K2b Knowledge and skills:** Nurse leaders were able to apply what they had learnt on the program in practice. Nurse leaders had a better understanding of themselves as leaders implementing change, assuming authority, supporting peer learning and compassionate care delivery.
Care home setting				
Dewar and	Several high-profile	AI, which has a	The focus was on what	**K2a Attitudes:** Staff generated positive

Reference	Context	Mechanism		Outcomes (intended or measured)
		Resource	Reasoning	
MacBride, 2017.	reports indicate that unacceptable standards of care for older people remain prevalent in the UK and internationally.<br><br>Development of interventions that focus on work-based educational models with real-time feedback, supporting people to engage in a way that demonstrates attunement, openness and curiosity is required.	unique focus on existing organizational strengths, rather than weaknesses, and the underlying assumption that people and organizations are full of assets, capabilities, resources and strengths that can be located, affirmed, leveraged and encouraged.	is working.<br><br>The research team provided immediate feedback to generate discussion on why particular interactions worked well. There were positive and encouraging interactions.<br><br>Photoelicitation with image cards. Participants (staff, residents, families) selected an image to sum up dignity and how they felt when communication worked well.<br><br>Positive inquiry tool which poses two affirmative questions: 'what is working well for you here?' and 'how can your experience be improved?' This was used to understand	caring practice statements and had an enhanced awareness of processes of skilled human interaction. Staff learned more about themselves and had increased confidence. Staff had a renewed sense of hope in moving forwards. There was enhanced individual and team morale.<br><br>**K3 Behaviors:** Positive interactions that mattered to staff, residents and families. Staff tried to connect emotionally more. Staff tied to be more curious about what mattered to the residents. Staff engaged in a process of inquiry about language they used. Staff consciously engaged in a 'new way' of interacting more of the time.

Reference	Context	Mechanism		Outcomes (intended or measured)
		Resource	Reasoning	
			people's experiences of interactions.<br><br>Discussion and asking questions.<br><br>Learning and understanding about others from their perspective.<br><br>Observing what is working well.<br><br>Teamwork.<br><br>Develop positive caring practice statements.<br><br>Connecting emotionally, sharing incidences when this happened, and recognizing that this could positively impact relationships encouraged staff to try to connect emotionally more of the time.	

Reference	Context	Mechanism		Outcomes (intended or measured)
		Resource	Reasoning	
			Required support from management, with a shared vision of the value of Caring Conversations in the workplace and a commitment to embed the processes into routine practice.	
Compassion training supporting individual healthcare provider level				
Acute care setting				
Stecker and Stecker, 2012.	The quality of patient care and maintenance of patient safety depends largely on the competence of nursing staff with respect to assessment skills and recognition of patients who are acutely ill to provide patient safety and favorable outcomes.<br><br>Nurses need to be provided with a structured educational program that both assesses their level of competence and	Evidence-based curriculum.<br><br>PowerPoint presentations to emphasize protocols.<br><br>Didactic.<br><br>Case-based scenarios.	Interaction between the nurse practitioner and participants.<br><br>Reinforcement of existing protocols to guide novice nurses in assessing patients.<br><br>Didactic material helped nurses apply old and new knowledge to plan their patient care.<br><br>Problem-based learning.<br><br>Engagement in simulation decision-making exercises.	**K2b Knowledge/skills:** Nurses showed improvements in areas of neurological examination and compassion/respect[†].

Reference	Context	Mechanism		Outcomes (intended or measured)
		Resource	Reasoning	
	provides specific education to improve their skills in recognizing patients who are acutely ill.		Multimodal process of education.	
Anderson et al., 2016.	Public report cards and value-based purchasing are driving new, patient-centered processes aimed at improving efficiency, safety, and the patient experience.<br><br>Health systems are becoming more open to exploring innovative and creative solutions to fulfill these aims, including nonpharmacological approaches to patient care.	Complementary therapy.<br><br>Training in a nonpharmacological, noninvasive intervention of blended traditional and contemporary healing practices.	Voluntary attendance.<br><br>Group sessions fostered support and encouragement among nurses.<br><br>Mentoring and sharing the techniques with coworkers and patients.<br><br>Holistic approach.	**K2a Attitudes:** Training grounded nurses making them calmer at work, more effective caring for patients and in helping coworkers stay calmer. Personal transformation in the nurses and their work. Nurses gained a better awareness of their role and participation in interacting with patients and on the impact of their training on the delivery of care.<br><br>**K2b Knowledge and skills:** Nurses shared their skills with coworkers and patients. Nurses modified the intervention to address time barriers, so it could be used during daily patient care. Nurses matched specific techniques to individual patients. Nurses used their training for self-care.<br><br>**K4b Benefits to patients:** Training improved pain and pain management, and nurse– patient interactions.
Palliative care				
Brown and Halupa, 2015.	Nurses working with dying patients with	Evidence-based curriculum.	Best practices in teaching and	**K1 Reactions:** Nurses agreed that it was important to incorporate a palliative care

Reference	Context	Mechanism		Outcomes (intended or measured)
		Resource	Reasoning	
	HIV should provide a peaceful death experience through compassionate care; however, this goal is not always achieved.<br><br>There is a lack of clinical experience, knowledge, and competency in discussion of end-of-life care issues when providing compassionate care to HIV/AIDS patients	Palliative care curriculum delivered in a traditional conference/ classroom	learning, including PowerPoint presentations, videos, handouts, and class participation.<br><br>Opportunities for critical thinking in regard to palliative care.<br><br>Nurses volunteered.<br><br>No CME credits, but nurses were paid for the educational course, and a certificate of completion was issued.	course in nursing orientation.<br><br>**K2a Attitudes:** Nurses felt they improved in providing palliative care to patients and in taking responsibility for their practice.<br><br>**K2b Knowledge and skills:** Nurses scored best on symptom management of patients with HIV/AIDS and scored lowest on basic information such as pathophysiology and drug therapy of patients with HIV/AIDS*.
Chan, 2018.	Nurses may lack education and knowledge in the skills and competencies necessary for providing quality end-of-life care to patients and families during the transition at the end of life.	Evidence-based curriculum.<br><br>The CARES Tool Comfort, Airway, Restlessness and delirium, Emotional and spiritual support, and Self-care. It is a pocket-size card reference that provides acronym-organized	Program facilitators.<br><br>Content delivery.	**K3 Behaviors** Participants were better able to communicate with patients, engage in active listening, and provide a supportive presence.

Reference	Context	Mechanism		Outcomes (intended or measured)
		Resource	Reasoning	
		prompts to address the most common symptom management needs of the dying.		
Betcher, 2010.	Nurses find discussions of emotional and spiritual issues with palliative care patients and families difficult and uncomfortable.<br><br>Lack of effective and compassionate communication is a barrier to planning care, developing mutually agreeable goals, and providing honest information while preserving hope.	Role-play and simulation.<br><br>Role-playing and simulation with actors from the University's School of Theatre and Film.<br><br>Didactic lecture.<br><br>Videotaping.<br><br>Debriefing.	Creation of a realistic environment through role playing with actors.<br><br>Concentrating on the emotional issues instead of physical assessment.<br><br>Replaying conversations and discussing with facilitators.<br><br>Videotaping allowed nurses to identify what they were doing to help the patient and family.<br><br>Reflection.<br><br>Emotional expression.<br><br>Self-awareness.	**K2a Attitudes:** Nurses had increased confidence in their ability to portray a caring attitude and develop a caring relationship with patients*.<br><br>**K2b Knowledge and skills:** The program helped nurses improve their communication skills with patients in palliative care and their families.

Reference	Context	Mechanism		Outcomes (intended or measured)
		Resource	Reasoning	
			Scenarios were appropriate for orienting new nurses and providing continuing education for nurses on communication.<br><br>Required a large amount of time and human resources to implement.	
Mental health setting				
Bunyan et al., 2017.	Recently, there has been a national outcry regarding poor care and lack of compassion in the UK NHS (Francis, 2013) and a vision of nursing, characterized by the '6C's' (care, compassion, competence, communication, courage and commitment), has been set out by the Department of Health (2012).	Evidence-based curriculum.<br><br>Listening skills training: using specific reflective listening skills, the clinician validates the patient's views, gently timing the eliciting and strengthening of 'change talk', whilst resisting the 'righting reflex' (the urge to 'fix' things through persuasion, advice etc., that is	Discussion.<br><br>Relevant to work.<br><br>2-day training was too short to elicit change.<br><br>Nurses working a 24-hour shift pattern were unable to access supervision sessions.	**K1 Reactions**: Nurses agreed or strongly agreed that the course achieved its objectives.<br><br>**K2b: Knowledge and skills:** Nurses had a better understanding of how to listen to patients and respect their autonomy whilst working with them to reach goals. Nurses had difficulty 'unlearning' habitual righting reflex responses as nurses made little attempt to understand the patient's point of view or develop a collaborative approach.<br><br>**K4 Patient benefits:** Patient experienced was improved but not in a sustained manner**.

Reference	Context	Mechanism		Outcomes (intended or measured)
		Resource	Reasoning	
	Locally, patient satisfaction surveys and serious incident reviews raised concerns about rehabilitation nurses' communication.	counterproductive for change).		
McEwan et al., 2020.	As poor wellbeing is associated with reduced quality of patient care, staff sickness, higher turnover rates and provides a barrier to compassionate care, there is a need for addressing burnout and poor wellbeing in mental healthcare professionals.	Contemplative therapy.<br><br>Compassionate Mind Training (CMT).	Delivered by the originator of CMT.<br><br>Manager's enthusiastic support for the approach was highly influential in allowing staff the time to implement it Approach was layperson-friendly and provided common examples that were easy to relate to.<br><br>Multi-media, participatory elements and humor.<br><br>Person-centered approach.<br><br>Adaptable program.	**K1 Reactions**: The course was positively evaluated by staff, characterizing it as 'thought-provoking', 'useful', 'helpful', and 'beneficial'. The course was engaging and enjoyable. Some nurses lacked confidence in practicing CMT, or the practices felt unnatural and embarrassing.<br><br>**K2a Attitudes:** Nurses reported the chance to 'slow down' and build on relationships with patients in what are usually fast-paced working environments. Nurses reframed scenarios in which they would normally engage in self-criticism. Nurses reported increased compassion and reduced criticism of colleagues and patients.<br><br>**K2b: Knowledge and skills:** Nurses had a better understanding of threatening behavior.

Reference	Context	Mechanism		Outcomes (intended or measured)
		Resource	Reasoning	
			Theory-heavy.<br><br>Lack of follow-up training or supervision.<br><br>Time constraints.<br><br>Organizational constraints: lack of support for staff training; the 'target-driven' culture that was perceived as nonconductive to a compassionate working; the individualistic culture of their organization; and the lack of emphasis on staff wellbeing.<br><br>Practice needed to be fully embedded in the team.	**K4 Patient benefits:** Nurses found sharing the approach with patients beneficial.
Oncology setting				
De Souza, 2014.	Focus on the importance of compassion in the delivery of nursing	Role play and simulation.<br><br>Reflection on	Role-play.<br><br>Experiential learning.	**K2a Attitudes**: Nurses were able to enter into a person's experience within their support system, develop an understanding of their burden, and to

Reference	Context	Mechanism		Outcomes (intended or measured)
		Resource	Reasoning	
	care has increased following the publication of the Francis report (Francis, 2013).	individuals' lives and illness experiences.	Reflection.<br><br>Discussion.<br><br>Scenario exploration increases theory practice links and relevance awareness.<br><br>Interactive learning.<br><br>Only relevant to small groups as resourcing is an issue when conducting the experience in a large cohort.<br><br>Not all educators are comfortable with these types of teaching methodologies.	share some of it with them.
Mixed settings				
Schneider et al., 2018<br>(Current or retired nursing professors).	There is a requirement for service learning within high-risk communities to improve access to healthcare for marginalized	Clinical instruction and community service.<br><br>Service learning.	Experiential learning.<br><br>Reciprocal learning.<br><br>Reflective journaling.<br><br>Group briefing discussions.	**K2b Knowledge and skills:** Nurses learned about the social determinants of health, development of compassion, appreciation of community nursing, professional growth and community engagement. Nurses developed specific nursing skills.

Reference	Context	Mechanism		Outcomes (intended or measured)
		Resource	Reasoning	
	persons.		Combination of clinical instruction with community service.<br><br>Institutional support by administration, faculty, and professors.<br><br>Nurses experienced moral distress.<br><br>Lack of sustainability due to resistance to community nursing in nursing education.<br><br>Lack of integration with other nursing courses.	
Hawthornthwaite et al., 2018.	There is a need for patient perspectives to be central to the planning, delivery, and evaluation of health care services.<br><br>Patient and family stories can generate valuable insight for practitioners into the	Reflective practice.<br><br>Patient storytelling.	Reflection.<br><br>Sharing and discussion of patient and family stories.<br><br>Educating and connecting with audiences.<br><br>Facilitators were key in	**K1 Reactions:** The curriculum was reported to be highly informative. The experience of hearing patient stories was “real,” “personal,” “honest,” or “relatable.” Patient perspectives had educational value, but nurses called for delivery of a broader range of stories and perspectives to further enrich their learning.<br><br>**K2a Attitudes**: Nurses were inspired to

Reference	Context	Mechanism		Outcomes (intended or measured)
		Resource	Reasoning	
	patient experience by evoking an emotional response, motivating listeners to reflect on their practice, and delivering relevant educational content.		creating a safe environment for interaction between participants and storyteller.	consider changing something in how they perform their job. Nurses had a much better understanding of the patients' and families' situation and needs.
Wiklund Gustin and Wagner, 2013.	Suffering is closely related to individuals' experience of alienation from others, a sense of being cut-off from community and experiencing oneself as inferior to others, and also contributes to perceived loss of self and dignity.<br><br>There is a need to develop self-compassion as it might be vital for avoiding compassion fatigue and promote compassion satisfaction to enable caregiver to deal with the other person in	Reflective practice.<br><br>Writing.<br><br>Dialogue.	Second researcher to support the first researcher's reflections on the sessions (validating reflections by matching experiences).<br><br>Personal and group hermeneutic reflections.<br><br>Dialectic dialogue.<br><br>Preunderstanding of suffering and compassion.<br><br>Putting pre-understanding aside to make room for new understandings.	**K2b: Knowledge and skills**: Nurses expanded their understanding of self-compassion and compassionate care.

Reference	Context	Mechanism		Outcomes (intended or measured)
		Resource	Reasoning	
	suffering.			
Brathovde, 2017 (emergency, medical-surgical, behavioral health, perioperative, and nursing administration).	There are increasingly complex challenges for nurses in the health-care setting.<br><br>This included adapting to changes in the healthcare culture, which creates unpredictable pressures.	Complementary therapy.<br><br>Self-care holistic practice (Reiki energy therapy).	Voluntary attendance<br><br>Introductory session several months before the actual training session.<br><br>Two Reiki Master registered nurses provided the training, and a registered nurse volunteer with Reiki Level II training assisted.	**K2a Attitudes**: Positive changes occurred in presence, self-care, spirituality, and personal attributes*. Nurses increased awareness of their own self-reflection and felt, or projected, a calm presence in the midst of a busy, stressful day. Nurses were more emotionally and spiritually present for their patients. Training brought nurses closer to their perception of their own faith and religious values. Nurses connected with their patients on a spiritual level. Nurses reported that taking care of themselves helped them take care of others. Nurses maintained benefit from training as they intended to pursue education in other healing arts modalities.
Mahon et al., 2017.	There is a need to address stress and burnout among nurses as stress, burnout, and compassion fatigue impact negatively on the caring relationship and the healing environment.	Contemplative therapy.<br><br>Instruction and practical training in mindfulness meditation techniques (6 and 8-week course).	Nurses had never attended an MBSR course or meditated and/or practiced mindfulness.<br><br>Patient care was priority, which can compromise attendance.<br><br>Cost and requisite time	**K2a Attitudes:** The intervention impacted positively on nurses' perceived stress and enhanced nurses' mindfulness and compassion levels*.

Reference	Context	Mechanism		Outcomes (intended or measured)
		Resource	Reasoning	
			commitment affected recruitment.	
Richards et al., 2006<br>(staff nurse, presurgical care, float pool, postsurgical orthopedic, pulmonary rehabilitation, and occupational health).	Changing hospital environments have added new stressors to the already demanding nursing profession, leading to increases in job-related burnout.<br><br>Staff shortages, interpersonal conflicts with coworkers, pressures from management, and increasing demands of patients and their families have been identified as some of the key stressors affecting the well-being of nurses and their ability to deliver care.	Contemplative therapy.<br><br>The Eight Point Program of Easwaran (EPP).<br><br>Spiritually based self-management tools.<br><br>Presentation.<br><br>Home assignments.<br><br>Group meditation.	"Passage meditation": concentrating on a memorized inspirational passage.<br><br>Physician instructor and co instructors skilled in EPP.<br><br>Participants functioned as models for each other in using program tools.<br><br>Program was accessible and useful, in the workplace.<br><br>Course credit and cash incentive.<br><br>Engaging with EPP practices.<br><br>Small group discussions.<br><br>Feedback.	**K1 Reactions:** Nurses questioned their abilities to sustain their practices on their own as the program was practice based. Nurses continued to apply several of the practices and principles 2 to 4-months following the intervention and were continuing to feel the positive impact of the practices on their well-being and their relationships within their work environments.<br><br>**K2a Attitudes:** Nurses reported enhanced personal capacities that were essential to workplace performance. Nurses felt a renewed sense of enjoyment in their work and more emotional balance at work. Nurses felt an increased ability to experience compassion and empathy. Nurses reported a new self-awareness that influenced their relationships.

Reference	Context	Mechanism		Outcomes (intended or measured)
		Resource	Reasoning	
			Time commitment created an obstacle to maintaining a daily meditation practice.	

Outcomes are intended and unintended consequences – K1 Reactions: Reactions and satisfaction with training (How much did they like it? How did participants react to it?); K2a Attitudes: Did attitudes change?; K2b Knowledge and skills: Did they learn anything? Did the authors use any established instruments to measure changes in knowledge; K3 Behaviors: Did the program or curriculum change their behaviors at all? Or future behaviors?; K4a Changes to clinical processes: Did the program or curriculum lead to any improvements to clinical processes; K4b Benefits to patients: Did the program or curriculum lead to any improvements to patients? (clinical outcomes; *outcomes on nurse self-report measures; ** outcomes on patient report measures; † external assessor (researcher, experience healthcare provider, peer) rated outcomes).

Contexts were defined as conditions in which compassion training was introduced and that triggered the training (background circumstances/ unmet need); mechanisms explained the impact of the component introduced by the context (the under lying resources) on the cognitive or emotional decisions and behaviors of the learners (reasoning) that caused compassion training to produce a change; and outcomes were defined as intended and/or unintended consequences of compassion training (Jolly and Jolly, 2014; Salter and Kothari, 2014; Dalkin et al., 2015).

Abbreviations: AI: Appreciative Inquiry, CFT: Compassion Focused Therapy, CLECC: Creating Learning Environments for Compassionate Care, CME: Continuing Medical Education, CMT: Compassionate Mind Training, EPP: The Eight Point Program of Easwaran, MBSR: Mindfulness-based stress reduction.

References

Department of Health (2012). Compassion in Practice. (London: Department of Health). Retrieved February 19, 2020, from https://www.england.nhs.uk/wp-content/uploads/2012/12/compassion-in-practice.pdf

Francis, R. (2013). Report of the Mid Staffordshire NHS Foundation Trust public inquiry. (London: The Stationary Office).

Kirkup, B (2015). The Report of the Morecambe Bay Investigation. (London: The Stationery Office). Retrieved February 19, 2020, from https://www.gov.uk/government/publications/morecambe-bay-investigation-report.

Clinicians: Context mechanism outcomes matrix by study

Reference	Context	Mechanism		Outcomes (intended or measured)
		Resource	Reasoning	
**CLINICIANS**				
Primary care				
Karkabi et al., 2014 (GPs/ Family physicians).	Reflective capacity is integral to core healthcare professional practice competencies.<br><br>Reflection is not necessarily intuitive for learners.<br><br>While both the arts and narrative can foster reflection and cultivate aesthetic ways of knowing within medical education, their combined use within a faculty development paradigm has not been reported in the literature.	Reflective practice.<br><br>Combined use of abstract paintings and narratives.	Use of art.<br><br>Viewing abstract paintings.<br><br>Writing and sharing narratives.<br><br>Reflection.	**K1 Reactions:** Viewing abstract paintings facilitated a valuable mood transformation and prepared participants emotionally for the reflective writing and the reflective exercise.<br><br>**K2aAttitudes:** Writing reflective narratives promoted compassion for self and compassion for others through recognition of shared humanity. Sharing the narrative promoted reflective self-assessment for personal and professional development.<br><br>**K3 Behaviors**: Sharing the narrative fostered active listening and appreciating multiple perspectives.
Verweij et al., 2016 (GPs).	Burnout has serious negative consequences not only for clinicians themselves, but also for patient care and clinical outcomes.<br><br>Burnout can lead to increased medical errors and reduced quality of patient care.	Contemplative practice.<br><br>Presentation, discussion, MBSR training and practice at home.	GPs were self–selected.<br><br>MBSR training was offered as part of a regular continuing professional development program and accredited by the professional bodies.	**K1 Reactions:** Most GPs stated that they learned and benefited from the training, but two GPs did not. GPs indicated the training was feasible and acceptable.<br><br>**K2aAttitudes:** Training decreased depersonalization and increased dedication*; increased awareness of

Reference	Context	Mechanism		Outcomes (intended or measured)
		Resource	Reasoning	
			GPs were allocated to the training period of their choice.<br><br>Taught by experienced trainers.<br><br>Weekly themes that were explicitly linked to the context of clinical care.<br><br>Supervising trainees at the start of the sessions also increased acceptance and facilitated learning.<br><br>Supportive environment (learning from one another).<br><br>Acceptance of thoughts and emotions to be able to put things into perspective.	bodily sensations, thoughts, emotions, beliefs, values and maladaptive patterns of behavior. Mindfulness training helped participants balance and harmonize with increased energy and joy in life. Participants experienced attitudinal changes towards themselves, aspects of self-acceptance and compassion. Mindfulness training taught participants to accept others, including their patients, and to have compassion for them.<br><br>**K2b Knowledge and skills:** Mindfulness skill increased.<br><br>**K3 Behaviors:** Participants made more deliberate choices, took rest, set limits; and took better care of themselves.
Schroeder et al., 2016 (Primary care).	Primary care physicians experience high rates of burnout, which results in diminished quality of life,	Contemplative therapy.<br><br>Mindful	Participants volunteered.<br><br>Participants had high baseline scores for	**K2a Attitudes:** The MMC resulted in significant improvements in stress, mindfulness, emotional exhaustion, and depersonalization 3-months after

Reference	Context	Mechanism		Outcomes (intended or measured)
		Resource	Reasoning	
	poorer quality of care and workforce attrition.<br><br>Burnout is linked to lower productivity, early retirement, and higher rates of turnover, which have profound financial impacts.<br><br>There is a need for evidence-based methods to reduce burnout and mitigate its negative impact among physicians.	Medicine Curriculum (MMC), which is a modified version of MBSR, with added elements of compassion skills training, brief mindfulness techniques designed to be used at work, and "SLO conversation" exercises where participants practice applying mindfulness to the core clinical skills of Speaking, Listening, and Observing (SLO).	patient self-reported satisfaction with their primary care physician.<br><br>Mindfulness training had an abbreviated format (a traditional 8-week MBSR course may be detrimental to enrollment).<br><br><10 min formal mediation practice outside the sessions was required to achieve improvements.<br><br>Group interactions.<br><br>Instructors with extensive experience in secular mindfulness-based interventions and familiarity with the culture of physicians.<br><br>Presented using secular, accessible language.<br><br>Program served as an introduction to	training; no significant improvement in resilience, compassion, or personal achievement*.<br><br>**K3 Behaviors**: Participants endorsed ongoing formal/informal meditation<br><br>**K4b Benefits to patients:** The MMC had no impact on patient-reported satisfaction with their primary care physician**.

Reference	Context	Mechanism		Outcomes (intended or measured)
		Resource	Reasoning	
			mindfulness that was relevant to the professional contexts in which physicians work.	
Fortney et al., 2013 (Primary care).	Burnout is more common among physicians than among other professionals, with specialties on the front lines such as primary care being at greatest risk.<br><br>Considerable evidence suggests that burnout negatively affects patient care.<br><br>Physicians tend to give suboptimal attention to self-wellness.	Contemplative therapy.<br><br>Meditation: An abbreviated version of the 8-week MBSR program.	Participants volunteered.<br><br>Participants had high compassion scores at baseline.<br><br>Training had secular and academic appeal, and a solid scientific foundation.<br><br>Training was low cost, collegial, and time efficient.<br><br>Participants were encouraged to view clinical work as an opportunity to practice mindfulness.<br><br>Mindfulness instructors were professionally trained.<br><br>Shared group setting.	**K2aAttitudes**: Significant reductions in burnout, depression, anxiety, and stress but no significant change resilience or compassion *; effect was maintained over 9-months.

Reference	Context	Mechanism		Outcomes (intended or measured)
		Resource	Reasoning	
			Dedicated Web site for the study that provided instruction.<br><br>Group effect.	
Palliative care				
Arnold et al., 2016.	The personal and clinical experiences of physicians, which are grounded in their relationship with their patients, remain understudied and the least understood for delivering optimal palliative care.	Reflective practice.<br><br>Visual narratives using paintings.	Participants were at the conclusion of a one-year palliative medicine fellowship.<br><br>Expression of thoughts and feelings through the arts and humanities.	**K2a Attitudes:** Physicians intended to integrate their clinical skills with their human skills.
Mental health				
Riches et al., 2019.	Simulation training is an increasingly widespread and effective teaching tool enabling learners to gain a subjective understanding of a range of skills.<br><br>Technology that can simulate psychotic experiences and increase understanding may address issues with staff stigma towards people having psychotic experiences.	Simulation.<br><br>Simulation of auditory hallucinations.<br><br>Voice recordings by professional actors.<br><br>Debriefing.	Participants were volunteers invited to attend an immersive art exhibition.<br><br>Group setting.<br><br>Professional actors performed voice “characterizations” developed in workshops with young people who hear voices.<br><br>Engaging clinical staff.	**K1 Reactions:** The simulations were acceptable and enjoyable; participants were motivated to partake in experiential learning in relation to auditory hallucinations and other psychotic experiences.<br><br>**K2a Attitudes**: Participants reported increases in understanding what it feels like to hear voices, compassion towards people who hear voices, and comfort talking to people who hear voices; current happiness decreased*.

Reference	Context	Mechanism		Outcomes (intended or measured)
		Resource	Reasoning	
			Tailoring voices to individuals.<br><br>Training conducted in an everyday setting but not a clinical setting.<br><br>Long duration.	

Outcomes are intended and unintended consequences – K1 Reactions: Reactions and satisfaction with training (How much did they like it? How did participants react to it?); K2a Attitudes: Did attitudes change?; K2b Knowledge and skills: Did they learn anything? Did the authors use any established instruments to measure changes in knowledge; K3 Behaviors: Did the program or curriculum change their behaviors at all? Or future behaviors?; K4a Changes to clinical processes: Did the program or curriculum lead to any improvements to clinical processes?; K4b Benefits to patients: Did the program or curriculum lead to any improvements to patients? (clinical outcomes?; *outcomes on physician self-report measures; ** outcomes on patient report measures).

Contexts were defined as conditions in which compassion training was introduced and that triggered the training (background circumstances/ unmet need); mechanisms explained the impact of the component introduced by the context (the under lying resources) on the cognitive or emotional decisions and behaviors of the learners (reasoning) that caused compassion training to produce a change; and outcomes were defined as intended and/or unintended consequences of compassion training (Jolly and Jolly, 2014; Salter and Kothari, 2014; Dalkin et al., 2015).

Abbreviations: GP: general practitioner, MBSR: mindfulness-based stress reduction, MMC: Mindful Medicine Curriculum

Multidisciplinary: Context mechanism outcomes matrix by study

Reference	Context	Mechanism		Outcomes (intended or measured)
		Resource	Reasoning	
**MULTIDISCIPLINARY**				
High-risk populations				
Chambliss et al., 1990.	Healthcare providers must become aware of their own attitudes arising about members of a high-risk population.	Evidence based curriculum.<br><br>Interactive staff training method including a presentation, videos and opportunity for asking questions.	Required attendance if adequate staffing of wards.<br><br>Sessions were scheduled to overlap shifts in order to accommodate staff needs and make participation more convenient.<br><br>Open discussion.<br><br>Interactive shared problem-solving strategy.<br><br>Training method seemed to circumvent an adversarial framework, where "management" is seen as presenting information as a means of coercing staff	**K2a Attitudes:** Participants reported improved compassion and acceptance of obligation to treat and appropriate work-related risk reduction*. Participants expressed a greater appreciation for the special needs of AIDS patients and greater compassion for the problems facing the asymptomatic HIV patient, but only if they completed both training sessions.

			compliance in an authoritarian, unresponsive manner.<br><br>Training sessions were designed for consecutive participation; therefore, those who had failed to attend the first session and only came to the second session were not expected to derive full benefit from the second session.	
Palliative care				
Moore et al., 2017.	There is a need for an integrated care approach for patients with dementia, where organizations and care professionals bring together all of the different elements of care that a person needs.<br><br>Providing good end of life (EOL) dementia care is complex, prognosis is unpredictable and managing symptoms is difficult when communication is compromised.	Leadership and team practices.<br><br>Weekly core meetings.<br><br>Education, training and support.<br><br>Discussions.	Interdisciplinary care leader (ICL) with a broad range of skills from the fields of nursing, social work or a profession allied to medicine.<br><br>ICL provided mentoring, role modeling, advice and training.<br><br>Time constraints to attend weekly meetings and training.	**K1 Reactions:** Training was positively evaluated by staff; the intervention topic was perceived to be of high importance.<br><br>**K2b Knowledge and skills**: Clinical knowledge was improved; staff developed new skills and ideas to improve care. The need for staff development and a shift from task-driven to compassionate care would require a longer duration and further training and support.<br><br>**K4b Benefits to patients:** The intervention did not cause harm to

	The need for a complex intervention is reflected in the European Association for Palliative Care's 57 recommendations for optimal EOL dementia care.		Implementation required a state of readiness for accepting the intervention with good external multidisciplinary support.	patients. The frequency of deaths, unplanned hospitalizations and out-of-hours calls was low.
Orellana-Rios et al., 2017.	There is a need to address the impact of stressors on the health and well-being of palliative care practitioners, as external factors including limited healthcare resources, increased clinical demands, and negative workplace cultures, can hinder the delivery of compassionate medicine.	Contemplative therapy.<br><br>"On the job" mindfulness and compassion-oriented meditation training (meditations and one-to one sessions, meditate at home, CD with guided exercises and posters with reminders to practice mindfulness).	Participants were volunteers at a faith-based community hospital. Participants could apply meditation instructions within the context of their own spiritual background.<br><br>Participants already had a compassionate attitude in their work.<br><br>"On the job" program, learners were instructed to apply the techniques at work.<br><br>Experienced meditation teacher and *Tong-len* expert.<br><br>Active participant involvement.	**K1 Reactions:** Participants were satisfied or partly satisfied with the course. They recognized the usefulness of the course, planned to implement these techniques into their work in the future and would recommend the course to other palliative care professionals.<br><br>**K2a Attitudes**: Reduction in perceived stress, anxiety and burnout and an increase in level of general joy; significant improvement in resilience and awareness*. No participant reported the training would have enhanced their compassion in general, but they wanted to underpin their professional competence in this area.<br><br>**K3 Behaviors:** Participants implemented self-care behaviors but few managed to optimize work routines. Interpersonal communication was enhanced.

			Opportunity to integrate practices in real-life situations and person-to-person interactions.<br><br>Self-awareness.<br><br>Training duration was short. The program was feasible and satisfactory for staff members in a busy unit.	
Rao and Kemper, 2017.	As hospitals and clinics are busy and intellectually demanding work environments, there is a need for meditation practices to improve cognitive function that could help clinicians process information more effectively and could improve patient outcomes.	Contemplative therapy.<br><br>Meditation practices: online modules, guided practice.	Online.<br><br>Cost-effective.<br><br>Convenient.<br><br>Feasible.<br><br>Self-reflection.	**K2a Attitudes:** Participants reported increased gratitude, well-being, self-compassion, and confidence in providing compassionate care to others *.
Mental health setting				
Suyi et al., 2017.	There is a need to reduce stress and burnout and promote positive attitudes in healthcare professionals.	Contemplative therapy.<br><br>Mindfulness program.<br><br>Discussion period	Monetary reimbursement facilitated recruitment.<br><br>Shortened program (6-weeks duration) to accommodate high	**K2a Attitudes:** Participants reported significant improvement in mindfulness, self-compassion, and compassion for others after a 6-week mindfulness program. Improvements in mindfulness and self-compassion scores were maintained 3-months later*. Stress

		to share stories.<br><br>Recordings of guided meditation for homework.	caseloads and tight schedules.<br><br>Experienced MBSR certified main instructor and co-facilitators.	was reduced after the program but this reduction was mot maintained for 3-months*. No change in burnout or disengagement*.
Elderly care				
Ross et al., 2013.	To improve the quality of care for older people deficits in specific knowledge, skills and attitudes of healthcare staff need to be addressed.	Role playing and simulation.<br><br>Human patient simulation in a high-fidelity simulation center and ward-based simulation exercises.<br><br>Role-playing.<br><br>Practical activities<br><br>Videotaping<br><br>Debriefing.<br><br>Discussions.	On the ward training.<br><br>Dedicated simulation training days with multiple time slots<br><br>Active learning and interactive participant-centered teaching.<br><br>Interprofessional group environment.<br><br>Clinicians and trained professionals facilitating discussions.<br><br>Reflection.<br><br>Immediate feedback from actors and team.<br><br>'Best practice' used during debrief	**K1 Reactions:** Program was well designed, delivered, facilitated and attended. Participants reported enjoyment of specific scenarios and exercises and were appreciative of the time and effort invested in them. Participants reported some anxiety and apprehension about the high-fidelity simulation.<br><br>**K2a Attitudes:** Participants confidence on key competencies increased*. Teamwork was strengthened.<br><br>**K3 Behaviors:** Participants perceived clearer communication with patients and relatives and between team members and improved provision of empathetic and supportive care.

			sessions.<br><br>Members of existing teams participated in training together (this had both positive and negative effects; participants felt challenged and some were reluctant to criticize).<br><br>No post program support from senior staff to maintain change.<br><br>Costs of the training need to be balanced against the organizational benefits and cost savings of increasing care quality.	
Farr and Barker, 2017.	Schwartz Rounds have not yet been studied within mental health and community services, and less research has been conducted on the implementation process of Rounds and the contextual enablers and constraints within organizations.	Rounds.<br><br>Rounds focusing on caregivers' experiences, encouraging staff to share insights, own their vulnerabilities,	Publicize.<br><br>Leaders and managers need to support Rounds. Senior managers need to endorse them and provide necessary resources. Team	**K1 Reactions:** Rounds were valued or seen as fluffy. Some leaders need evidence of value in relation to targets such as reductions in staff sickness.<br><br>**K2a Attitudes:** Participants are more patient aware, have improved communications with patients, and are more mindful of the emotional

		and support each other.	leaders must enable different staff to attend, supporting workload management.<br><br>Discussion enabling emotional resonance across interdisciplinary colleagues.<br><br>Reflection.<br><br>Facilitation, but senior managers as facilitators may make people less likely to open up.<br><br>Focus should be on emotional aspects, but detailed case study can lessen engagement.<br><br>Lack of staff time to attend, fears of exposing potential vulnerabilities in front of colleagues, and not being clear on what they would get out of attendance.	impact of work, alongside being more empathetic and compassionate.<br><br>**K3 Behaviors:** Better able to manage patients and work as a team.

			Where staff are more dispersed, costs of staff travel and time spent attending Rounds will be higher.	
Gale et al., 2017.	Personal practice in Compassion Focused Therapy (CFT) has a number of benefits, both for the therapist personally and for acting as a therapist.	Contemplative therapy.<br><br>CFT including personal practice.	Reflection.<br><br>Training was considered too short.	**K1Reactions:** The exercises were powerful; however, not having a CFT supervisor to be able to share the learning experience with, and to support participants in introducing it in clinical practice, was found by participants to have limited the potential benefits.<br><br>**K2a Attitudes:** Improved compassion for self and others, particularly clients.
Mixed settings				
Altamirano-Bustamante et al., 2013.	There is the need to promote career-long competence with respect to medical advances (evidence-based medicine); and fine-tuning of professional values and principles (values-based medicine) through continuing medical education (CME).	Evidence-based curriculum.<br><br>Clinical ethics course.	60-hour CME certification.<br><br>Free.<br><br>Online.<br><br>Designed by a cross-functional group (including medical doctors, teachers, anthropologists, sociologists, philosophers and bioethicists).<br><br>Motivational videos.	**K2a Attitudes:** Improved the high-order values of openness to change and self-transcendence*, justice, autonomy, love, charity compassion, and beneficence.

			Pictures.<br><br>Real-time decision-making.	
Reynolds et al., 2019.	There is a requirement for professional training and personal development to equip clinicians with the skills or attitudes to offset patient disengagement that can occur during challenging patient presentations.	Vignettes.<br><br>Online survey.<br><br>Imagery.	Social media and medical networks for recruitment.<br><br>Prize incentive.	**K2a Attitudes:** Compassion was induced*. There was less engagement with patients who were responsible for their illness and who presented with aversive symptoms*.
Han and Kunik, 2017.	It is unknown whether and how staff who attend Compassionate Touch (CT) coach training use the program for residents in their own settings, whether and how they train other staff in their own settings, and what potential benefits and barriers they encounter while using the program for residents and training other staff.	Complementary therapies.<br><br>CT: person-centered approach and touch protocol.	Leadership staff needed to lead by example.<br><br>Hands-on practice.<br><br>Video-recorded CT training material and handouts.<br><br>Scheduling issues.<br><br>Staff were paid to train.<br><br>Monthly training and an annual refresher.<br><br>Staff were uncomfortable with	**K1 Reactions:** The CT program was easy to learn and use and participants felt confident, comfortable, satisfied, and pleasant about using the program. Staff were hesitant and uncomfortable using the CT program in the clinical setting and had difficulty in finding the time for it.<br><br>**K3 Behaviors:** More than 95% of participants used CT with residents with dementia after training and 50% used CT fairly or very often. About 83% trained other staff in their settings after they attended the CT coach training.<br><br>**K4b Benefits to patients**: Using the CT program was beneficial for residents with dementia; reported

			the program.<br><br>Staff were resistant as 'it's not part of the job'.	benefits included calming and redirecting residents, decreasing residents' behavioral issues, and improving residents' mood and increasing connection between residents and staff.
Penson et al., 2010.	Experienced practitioners regularly emphasize the significance of the patient–caregiver relationship when they refer to the art of healing.	Rounds.<br><br>Rounds focusing on caregivers' experiences, and encouraging staff to share insights, own their vulnerabilities, and support each other.	Brief presentation (10 minutes).<br><br>Discussion.<br><br>Reflection.<br><br>Clear leadership from physician leaders and facilitators to encourage comments and questions and involve the whole group.<br><br>"Safe" place with the security that allows people to be candid.<br><br>Multidisciplinary meeting, with a nonhierarchical atmosphere and a level playing field.<br><br>Respectful and nonjudgmental	**K2a Attitudes:** Positive impact on staff morale; rekindled a sense of vocation or mission; fostered self-awareness and self-reflection.<br><br>**K3 Behaviors:** Facilitated effective teamwork.

			atmosphere.<br><br>Warm, welcoming atmosphere with food.<br><br>Sense of community and a vital common goal.	
Kemper and Hill, 2017.	There is a need to provide professional training in complementary therapies to address burnout.	Complementary therapies.<br><br>Training in a combination of complementary therapies.	Marketing was used to advertise the training.<br><br>CME credit.<br><br>Supportive peer learning environment.<br><br>Opportunity to ask questions.<br><br>Sustainable, enrollment for 3 out of 4 topics well above the calculated minimal number for financial viability.<br><br>Training offered by experienced clinician-teachers.<br><br>Learners register in greatest numbers for topics they have	**K1 Reactions:** Participants reported thatthe training met learning objectives, was well organized, that the training provided an opportunity to ask questions, and that they would recommend the training to other health professionals.<br><br>**K2a: Attitudes**: Participants reported that as a result of the training they planned to make changes in their personal self-care and their care of others*.<br><br>**K3 Behaviors:** There was a significant decrease in the percentage of participants who had unplanned work absences.

			requested.<br><br>Some resistance from some medical staff members to giving institutional ‘‘approval’’ to Reiki training.	
Kemper et al., 2017.	There is a need to address the stress, burnout, and depression that is increasingly reported by health professionals.	Contemplative practices.<br><br>Mind-body therapies: online course.	Offered free of charge to health professionals and trainees at the university; others were charged a nominal fee.<br><br>Elective, no course credit.<br><br>Online.<br><br>No required minimum number of units.<br><br>No deadline for completing the course.	**K3 Behaviors:** One year after the course, healthcare professionals reported changes in behavior associated with self care and caring for others*.
Moffatt-Bruce et al., 2019.	There is a need to identify and dedicate resources for maintaining and improving wellness and resilience among front line providers to assure quality of patient care.	Contemplative practices.<br><br>Multiprofessional, multimodal process and programs for introducing and sustaining	Organization and funding support.<br><br>Committed leadership.<br><br>Partnership with the creator of the mindfulness program	**K1 Reactions:** The program was well accepted and appreciated. Participants valued being taught breathing techniques.<br><br>**K2a Attitudes:** Participants reported significant improvements in confidence in providing calm, compassionate care self-compassion, at 8-weeks post-

		mindfulness training.<br><br>Online modules; interactive lecture series; didactic; guided meditation practice.	Feasible and cost-effective.<br><br>Interactive training with co-workers on the participants' unit, while clinical work was covered with float health care providers.<br><br>Booster sessions after the initial intervention offered at various times and supplemented with a meal.<br><br>Weekly reminders of mindfulness tips send by email.	baseline, and in perceived stress, depersonalization, interpersonal self-transcendence, and work engagement at 6-months post-baseline*. Participants appreciated the difference in reacting versus responding to an event, and the value of examining what drives one's work meaning.<br><br>**K4 Changes to clinical processes:** Cultural transformation around patient safety (significant reduction in the number of avoidable events).<br><br>**K4b Benefits to patients**: There were reduced safety events but no significant differences or decreases in participant self-reported patient satisfaction scores*.

Outcomes are intended and unintended consequences – K1 Reactions: Reactions and satisfaction with training (How much did they like it? How did participants react to it?); K2a Attitudes: Did attitudes change?; K2b Knowledge and skills: Did they learn anything? Did the authors use any established instruments to measure changes in knowledge; K3 Behaviors: Did the program or curriculum change their behaviors at all? Or future behaviors?; K4a Changes to clinical processes: Did the program or curriculum lead to any improvements to clinical processes?; K4b Benefits to patients: Did the program or curriculum lead to any improvements to patients? (clinical outcomes; *outcomes on healthcare provider self-report measures; ** outcomes on patient report measures).

Contexts were defined as conditions in which compassion training was introduced and that triggered the training (background circumstances/ unmet need); mechanisms explained the impact of the component introduced by the context (the under lying resources) on the cognitive or emotional decisions and behaviors of the learners (reasoning) that caused compassion training to produce a change; and outcomes were defined as intended and/or unintended consequences of compassion training (Jolly and Jolly, 2014; Salter and Kothari, 2014; Dalkin et al., 2015).

Abbreviations: CFT: Compassion Focused Therapy, CME: Continuing Medical Education, CT: Compassionate Touch, EOL: end of life, ICL: interdisciplinary care leader.
