# Supplementary material for: What works for whom in compassion training programs offered to practicing healthcare providers: a realist review
Source: BMC Med Educ. 2021 Aug 28;21:455. doi: 10.1186/s12909-021-02863-w (PMC8403363; doi:10.1186/s12909-021-02863-w)
Supplement: Supplementary file 5 — Additional file 5. Summary of Contexts, Mechanisms, and Outcomes. [file 12909_2021_2863_MOESM5_ESM.pdf]

Additional file 5: Summary of Contexts, Mechanisms, and Outcomes

**Contexts**		**N***	**References**
Setting.	Mixed.	14	64; 65; 70; 79; 80; 81; 82; 85; 98; 99; 101; 103; 105; 107;
	Acute care.	11	18; 67; 68; 69; 70; 71; 72; 88; 93; 94; 95;
	Palliative care.	7	77; 86; 89; 90; 91; 100; 106;
	Primary care.	4	76; 78; 87; 102;
	Mental health.	4	74; 83; 84; 97;
	Elderly care.	3	66; 92; 104;
	High-risk populations.	1	96;
	Care home.	1	73;
	Oncology.	1	75;
Healthcare provider.	Nurse.	24	18; 65; 67; 68; 69; 70; 71; 72; 73; 74; 75; 81; 82; 84; 88; 89; 90; 91; 93; 94; 95; 98; 101; 103;
	Clinician.	6	76; 78; 87; 97; 100; 102;
	Multidisciplinary.	15	64; 66; 77; 79; 80; 83; 85; 86; 92; 96; 99; 104; 105; 106; 107;
Healthcare system contexts.	Need for an integrated approach to care.	20	18; 67; 68; 69; 70; 71; 72; 73; 74; 75; 88; 92; 94; 95; 98; 101; 104; 105; 106; 107;
Healthcare provider contexts.	Need to nurture innate compassion.	16	64; 66; 74; 75; 89; 90; 91; 96;97; 99; 100; 101; 102; 103; 104; 105;
	Need for stress-reduction.	15	65; 66; 76; 77; 78; 79; 80; 81; 82; 83; 84; 85; 86; 87; 104;
	Need to improve clinical competence.	7	74; 90; 91; 92; 93; 97; 99;
**Mechanisms**		**N***	**References**
Resource			
Work-based educational models.	Development of leadership and team practices.	12	18; 67; 68; 69; 70; 71; 72; 73; 92; 94; 95; 106;
Participatory interventions supporting individual healthcare providers.	Professional development.	17	*Evidence-based curricula:* 74; 90; 91; 93; 96; 107;<br>*Clinical instruction and community service:* 98;<br>*Role playing and simulation:* 75; 89; 97;<br>*Vignettes:* 99;<br>*Reflective practices:* 100; 101; 102; 103;<br>*Complementary therapies:* 64; 88;
	Coping and resilience skills.	17	*Rounds:* 104; 105;<br>*Complementary therapies:* 65; 79; 88;

			Contemplative therapies: 66; 76; 77; 78; 80; 81; 82; 83; 84; 86; 87; 85;
Reasoning			
Teaching mechanisms.	Highly qualified program leaders and facilitators.	28	*Facilitator:* 18; 65; 67; 68; 69; 71; 72; 73; 76; 77; 78; 79; 82; 83; 84; 85; 87; 91; 92; 93; 94; 95; 97; 101; 105; 106; 107;<br><br>*Barrier:* 104;
	Team learning activities.	15	*Facilitator:* 18; 67; 68; 69; 70; 71; 72; 73; 85; 88; 92; 94; 95;<br><br>*Barrier:* 72; 92;
	Continuous learning.	36	*Facilitator:* 18; 64; 65; 67; 68; 69; 70; 71; 72; 73; 77; 78; 79; 80; 81; 82; 85; 87; 88; 94; 95; 106;<br><br>*Barrier:* 18; 64; 66; 67; 68, 74; 82; 84; 92; 94; 96;104; 106;
	Staff empowerment.	17	*Facilitator:* 18; 67; 68; 69; 70; 71; 72; 73; 76; 77; 85; 94; 95; 106;<br><br>*Barrier:* 67; 68, 94;
	Education in context.	22	*Facilitator:* 18; 67; 68; 69; 70; 71; 72; 73;74; 77; 78; 82; 87; 89; 93; 94; 95; 98; 104; 106;<br><br>*Barrier:* 18; 104;
	Contemplative practices.	13	*Facilitator:* 66; 76; 77; 78; 80; 81; 82; 83; 84; 85; 86; 87;<br><br>*Barrier:* 84;
	Discussion.	25	*Facilitator:* 18; 67; 68; 69; 70; 71; 72; 73; 74; 75; 82; 83; 87; 89; 92; 94; 95; 96; 97; 98; 103; 104; 105; 106;<br><br>*Barrier:* 92;
	Case-based scenarios.	2	*Facilitator:* 91; 93;
	Simulation and role play.	9	*Facilitator:* 75; 89; 92; 93; 97; 99;<br><br>*Barrier:* 75; 89; 92;
	Patient storytelling.	1	*Facilitator:* 101;
	Didactic lectures.	4	*Facilitator:* 85; 90; 93; 96;
	Video/audio taping.	3	*Facilitator:* 89; 92; 97;

	Multimodal approach.	8	*Facilitator:* 64; 84; 85; 90; 93; 96; 98; 107;
Learning mechanisms.	Group participation.	16	*Facilitator:* 18; 69; 70; 71; 72; 73; 76; 78; 79; 82; 87; 88; 92; 97; 103; 105;
	Feedback.	11	*Facilitator:* 18; 67; 68; 69; 70; 72; 73; 82; 92; 94; 95;
	Recognition and sharing of positive practices.	3	*Facilitator:* 70; 72; 73;
	Reflection.	24	*Facilitator:* 18; 66; 67; 68; 69; 70; 71; 72; 73; 75; 86; 89; 92; 94; 95; 98; 101; 102; 103; 104; 105;<br><br>*Barrier:* 76; 97; 104;
	Discussion.	25	*Facilitator:* 18; 67; 68; 69; 70; 71; 72; 73; 74; 75; 82; 83; 87; 89; 92; 94; 95; 96; 97; 101;103; 104; 105; 106;<br><br>*Barrier:* 92;
	Mentoring.	2	*Facilitator:* 88; 106;
	Experiential/hands-on learning.	8	*Facilitator:* 64; 69; 70; 71; 75; 92; 97; 98;<br><br>*Barrier:* 64; 75;
	Critical thinking and problem solving.	7	*Facilitator:* 69; 70; 71; 75; 90; 93; 96;<br><br>*Barrier:* 75;
	Linking theory to practice.	4	*Facilitator:* 69; 70; 71;<br><br>*Barrier:* 84;
Organizational mechanisms.	Integration of activities with the priorities of the wider organization.	17	*Facilitator:* 18; 69; 72; 73; 84; 85; 98;<br><br>*Barrier:* 64; 67; 68; 70; 79; 84; 94; 95; 104; 106;
	Time efficient.	10	*Facilitator:* 76; 77; 78; 83; 86; 87; 96; 104;<br><br>*Barrier:* 81; 106;
	Flexibility in programing.	6	*Facilitator:* 79; 80; 84; 92; 107;<br><br>*Barrier:* 64;
	Cost effective.	8	*Facilitator:* 76; 79; 85; 86; 107;<br><br>*Barrier:* 81; 92; 104;

	Course credits and cash/prize incentives.	9	*Facilitator:* 64; 79; 82; 83; 87; 90; 99; 107;<br><br>*Barrier:* 80;
**Outcomes****		**N***	**References**
Program outcomes.	K1 Reactions.	23	64; 66; 67; 68; 69; 70; 71; 74; 77; 79; 82; 84; 85; 87; 90; 92; 94; 95; 97; 101; 102; 104; 106;
Healthcare provider outcomes.	K2a Attitudes.	36	18; 65; 66; 67; 68; 69; 70; 71; 73; 75; 76; 77; 78; 79; 81; 82; 83; 84; 85; 86; 87; 88; 89; 90; 92; 94; 95; 96; 97; 99; 100; 101; 102; 104; 105; 107;
	K2b Knowledge and skills.	13	69; 70; 71; 74; 84; 87; 88; 89; 90; 93; 98; 103; 106;
	K3 Behaviors.	15	18; 64; 72; 73; 77; 78; 79; 80; 85; 87; 91; 92; 102; 104; 105;
Healthcare system outcomes.	K4a Changes to clinical processes.	1	85;
	K4b Benefits to patients.	11	64; 67; 68; 74; 78; 84; 85; 88; 94; 95; 106;

*N= number of references

**Outcomes are intended and unintended consequences – K1 Reactions: Reactions and satisfaction with training (How much did they like it? How did participants react to it?); K2a Attitudes: Did attitudes change?; K2b Knowledge and skills: Did they learn anything? Did the authors use any established instruments to measure changes in knowledge; K3 Behaviors: Did the program or curriculum change their behaviors at all? Or future behaviors?; K4a Changes to clinical processes: Did the program or curriculum lead to any improvements to clinical processes; K4b Benefits to patients: Did the program or curriculum lead to any improvements to patients?
