# Supplementary material for: What works for whom in compassion training programs offered to practicing healthcare providers: a realist review
Source: BMC Med Educ. 2021 Aug 28;21:455. doi: 10.1186/s12909-021-02863-w (PMC8403363; doi:10.1186/s12909-021-02863-w)
Supplement: Supplementary file 6 — Additional file 6. Learners’ characteristics. [file 12909_2021_2863_MOESM6_ESM.pdf]

Additional file 6: Learners' characteristics

Study	Number of participants (N)	Gender n(%)	Age (years)	Race n(%)	Qualification/Job title n(%)	Years in practice	Previous experience related to the program	Other
**NURSES**								
Workplace based programs								
Acute care setting								
Bridges et al., 2019.	"Boost" version of the Creating Learning Environments for Compassionate Care (CLECC1 Boost) N=42 questionnaires completed pre-intervention from 2 Wards Ward D N=25, Ward E N=17 N=28 post intervention Ward D N=10 Ward E N=18 "Enhanced" version (CLECC2) N=34 questionnaires completed pre intervention from 2 Wards Ward G N=12 Ward H N=22 N=11 post-intervention	CLECC1Boost Pre-intervention Female n=40 (95) Male n=1(2) Post intervention Female n=27 (96) Male n=1(4) CLECC2 Pre-intervention Female n=30 (88) Male n=4(12) Post Intervention Female n=10(91) Male n=1(9).	CLECC1 Boost Pre-intervention 25 or under n=5(12) 26-35 n=10(24) 36-45 n=13(31) 46-55 n=7(17) 56 or over n=6(14) Post intervention 25 or under n=2(7) 26-35 n=7(25) 36-45 n=7(25) 46-55 n=7(25) 56 or over n=5(18) CLECC2 Pre-intervention 25 or under n=5(15) 26-35 n=11(32) 36-45 n=11(32) 46-55 n=4(12) 56 or over n=3(9) Post intervention 25 or under n=3(27) 26-35 n=2(18) 36-45 n=2(18) 46-55 n=2(18) 56 or over n=1(9).	CLECC1 Boost Pre-intervention White British n=29 (69) Any other white n=1(2) White and black Caribbean n=1(2) White and black African n=1(2) Indian n=3(7) Any other Asian n=3(7) Other black/African/Caribbean n=1(2) Post intervention White British n=23 (82) Indian n=1(4) Any other Asian n=2 (7) Caribbean n=1(4) CLECC2 Pre-intervention White British n=22(65) Any other white n=4(12) White and Asian n=1(3) Indian n=4(12) Any other Asian n=1(3) Post intervention White British n=8(72) Any other white n=1(9) Any other Asian n=1(9).	CLECC1 Boost Pre-intervention Health care assistant n=18(43) Staff nurse n=14(33) Sister/Charge nurse n=4(10) Other n=6(14) Post intervention Health care assistant n=16(57) Staff nurse n=10(36) Sister/Charge nurse n=1(4) Other n=1(4) CLECC2 Pre-intervention-Health care assistant n=15(44) Staff nurse n=10(29) Sister/Charge nurse n=6(18) Other n=3(9) Post intervention Health care assistant n=5(45) Staff nurse n=3(27) Sister/Charge nurse n=2(18).	NR	CLECC1 Boost Few participants had been present for the original CLECC training CLECC2 No participant had previous involvement with CLECC training.	CLECC1 Boost Years on study ward Pre-intervention Ward D N=16 Mean 3(SD3) Ward E N=13 Mean 4(SD3) Post intervention Ward D N=9 Mean 4(SD2) Ward E N=17 Mean 5(SD4) CLECC2 Pre intervention Ward G Mean 0.7(SD0.7) Ward H Mean 1(SD0.8) Post intervention Ward G Mean 2(SD2) Ward H Mean 1(SD1).

Study	Number of participants (N)	Gender n(%)	Age (years)	Race n(%)	Qualification/Job title n(%)	Years in practice	Previous experience related to the program	Other
	Ward G N=3<br>Ward H N=8.							
Bridges et al., 2018.	N=86 questionnaires completed post intervention N=34 from the control ward, N=52 from the intervention ward.	Control ward<br>Female n=30(88)<br>Male n=4(12)<br>Intervention ward<br>Female n=43(83)<br>Male n=9(17).	Control ward<br>25 or under n=9(27)<br>26-35 n=11(32)<br>36-45 n=7(21)<br>46-55 n=5(15)<br>56 or over n=2(6)<br>Intervention ward<br>25 or under n=11(21)<br>26-35 n=14(27)<br>36-45 n=13(25)<br>46-55 n=8(15)<br>56 or over n=6(12).	Control ward<br>White British n=19(58)<br>Other white n=7(21)<br>Intervention ward<br>White British n=29(58)<br>Other white n=9(18).	Control ward<br>Healthcare assistant n=16(47)<br>Staff nurse n=13(38)<br>Sister/charge nurse n=4 (12)<br>Other n=1(3)<br>Intervention ward<br>Health care Assistant n=22(45)<br>Staff nurse n=21(43)<br>Sister/charge nurse n=4(8)<br>Other n=2(4).	Control ward<br>Mean 10(SD9)<br>Range 1-30<br>Intervention ward<br>Mean 10(SD8)<br>Range 0-30.	NR	
Bridges et al., 2017.	N=47 staff interviewed.	NR	NR	NR	Ward managers n=4<br>Deputy ward managers n=2<br>Staff nurses n=8<br>Healthcare assistants n=7<br>Senior hospital nurses n=2<br>Senior practice development nurses n=2.	NR	NR	Ward based interviewees had worked on their current ward for an average of 4 years. Range 2 weeks -14 years.
Bridges and Fuller, 2015.	Descriptive paper	NR	NR	NR	NR	NR	NR	The paper proposes and describes the use of the Creating Learning Environments for Compassionate Care (CLECC).
Dewar and Cook, 2014.	N=86	NR	NR	NR	Associate directors of nursing n=2<br>Clinical nurse managers n=5<br>Charge nurses/ward	NR	NR	Staff covered 24 inpatient areas

Study	Number of participants (N)	Gender n(%)	Age (years)	Race n(%)	Qualification/Job title n(%)	Years in practice	Previous experience related to the program	Other
					managers n=23<br>Senior staff/registered nurses n=23<br>Staff/registered nurses n=33.			
Dewar and Mackay, 2010.	Descriptive paper involving, staff, students, patients and families.	NR	NR	NR	NR	NR	NR	This paper used appreciative action research to explore, develop and articulate strategies that enhanced compassionate relationship centered care in an acute hospital setting, caring for older people.
Saab et al., 2019.	N=79.	Female n=78(99)<br>Male n=1(1).	Mean =46.1(SD6.9)<br>Range 32-60	NR	Clinical nurse/midwife manager n=73 (93)<br>Clinical nurse/midwife specialist n=3(4)<br>Assistant director of nursing/midwifery n=2(3)<br>Advanced nurse/midwife practitioner n=1(1).	Years qualified Mean =23.5(SD7)<br>Range 8-40.	NR	Clinical area n(%)<br>Obstetrics and gynecology n=15(19)<br>Surgical n=11(14)<br>Medical n=9(11)<br>Hematology/oncology n=7(9)<br>Emergency care n=6(8)<br>Orthopedics n=5(6)<br>Others n=26(33).
MacArthur et al., 2017.	Subjects - receiving the Leadership in Compassionate Care Program (LCCP) n=14<br>Practitioners - translating the program theories into practise n=7<br>Policy makers - influencing the direction of	NR	NR	NR	Subjects were charge nurses and nurse managers n=14<br>Practitioners were senior nurses n=7<br>Policy makers were senior individuals in the NHS organization and higher education institution n=5.	NR	Data regarding previous experience of subjects undergoing the program was not recorded.	The paper was a critical analysis of the impact of the LCCP using the perspectives of three stakeholders, subjects, practitioners and policy makers.

Study	Number of participants (N)	Gender n(%)	Age (years)	Race n(%)	Qualification/Job title n(%)	Years in practice	Previous experience related to the program	Other
	the program n=5.							
Landers et al., 2020.	N=15.	Female n=14(93) Male n=1(7).	Mean=49 Range 35-63.	NR	Clinical nurse/midwifery manager n=6 Director of nursing/midwifery n=4 Chief director of nursing/midwifery n=3 Program facilitator n=2 Highest academic qualification Bachelor's degree n=5 Higher/ Postgraduate Diploma n=4 Diploma n=2 Masters n=2 Doctorate n=2.	Mean 22(Range13-41).	The clinical nurse/midwifery managers had completed the program n=6.	The paper was an exploration of participants' views of the LCCP program from the perspective of nursing/midwifery leaders, directors of nursing/midwifery, chief directors of nursing/midwifery, and program facilitators who delivered the program. Current area of practice Administration n=7 Education n=2 Emergency department n=2 Clinical practice development n=1 Haematology Unit n=1 High Dependency Unit n=1 Maternity services n=1.
Care home setting								
Dewar and MacBride, 2017.	N=37 A core group of N=6 staff led the study with the researchers.	NR	NR	NR	Core group N=6, Care home manager n=1, Registered nurses n=2, Senior carers/activity coordinators n=3.	NR	NR	
Compassion training supporting Individual Healthcare Provider level								
Acute care setting								
Stecker and Stecker, 2012.	N=25.	Female n=20 (81) Male n=5 (19).	Mean=49 Range 24-62.	NR	Registered nurse n=21 (84) Licensed practice nurse n=4 (16)	Range of Experience 0-15 years.	NR	

Study	Number of participants (N)	Gender n(%)	Age (years)	Race n(%)	Qualification/Job title n(%)	Years in practice	Previous experience related to the program	Other
					BSc n=3 (14)<br>Associate Degree n=21 (86).			
Anderson et al., 2016.	Focus group N=11<br>Online narratives group N=24.	Female n=35(100%).	Focus group mean =51.3<br>Online narratives group mean =48.25.	Focus group Caucasian n=9(82)<br>Online narratives Caucasian n=21(88).	Focus group in nursing<br>Associate degree n=3 (27)<br>Diploma n=3 (27)<br>BSc n=5 (46)<br>Online narratives group<br>Associate degree n=5 (21)<br>Diploma n=5 (21)<br>BSc n=9 (37)<br>MSc n=4 (17)<br>Doctorate n=1 (4).	Focus group Mean = 24.5<br>Online narratives group<br>Mean = 23.25.	NR	Worked >36 hours per week n(%)<br>n=8(73) of the focus group<br>n=19(79) of the online narrative group.
Palliative care								
Brown and Halupa, 2015.	N=30	NR	NR	NR	Registered nurses who worked in critical care.	Nursing experience<br>0-3 years n=5<br>4-6 years n=4<br>7-10 years n=15<br>10 years or more n=6.	NR	At least 3 months of critical care experience.
Chan, 2018.	N=24.	Female n=19(79%)<br>Male n=5(21%).	Mean age 40.9(SD11.7)<br>Range 26-64<br>20-29 n=5(20.8)<br>30-39 n=7(29.2)<br>40-49 n=6(25)<br>50-59 n=3(12.5)<br>60-69 2 n= (8.4).	Caucasian n=13(54)<br>Asian/Native Hawaiian/Pacific Islander n=8(33)<br>Hispanic n=3(13%).	Registered nurses n=24(100%)<br>Associate Degree in Nursing n=7(29)<br>BSc Nursing n=15 (63)<br>Masters n=2(8).	NR	Had basic end of life training 10 (41.7%)<br>Used the CARES Tool 1 (4.2%).	n(%)<br>n=12(50) worked on medical units,<br>n=11(46) in critical care units<br>n=1(4) in ambulatory care.
Betcher, 2010.	N=8.	NR	NR	NR	NR	NR	NR	
Mental health setting								
Bunyan et al., 2017.	N=60.	NR	NR	NR	Nurses, healthcare assistants (HCA), psychiatrists, clinical psychologists,	NR	NR	

Study	Number of participants (N)	Gender n(%)	Age (years)	Race n(%)	Qualification/Job title n(%)	Years in practice	Previous experience related to the program	Other
					occupational therapists, social workers and administrators. As 82% of staff who attended the training days were nurses and the supervision days were attended by nurses (42%) and HCA (40%) the paper focuses on nursing practices.			
McEwan et al., 2020.	N=28 took part in training N=17 attended a focus group.	In the focus group Female n=12 Male n=5.	NR	NR	Mental health professionals.	NR	NR	
Oncology setting								
De Souza, 2014.	Descriptive paper.	NR	NR	NR	NR	NR	NR	Describes Family Sculpting
Mixed settings								
Schneider et al., 2018.	N=19.	Nursing professors n=10 Female n=10<br><br>Students n=9 Female n=7 Male n=2.	NR	NR	Nursing professors Nursing students.	Nursing students n=9 Undergraduate n=6 Graduates N=3.	NR	Nursing professors Full time n=7 Part time n=2 Retired n=1 Had supervised community practicums including nursing management, sexual health, occupational health, health promotion and/or adult care. Students Practicums: Occupational health n=1, adult care n=2, sexual health n=2, nursing management

Study	Number of participants (N)	Gender n(%)	Age (years)	Race n(%)	Qualification/Job title n(%)	Years in practice	Previous experience related to the program	Other
								n=3, Health promotion n=3.
Hawthornthwaite et al., 2018	N=542.	NR	NR	NR	NR	NR	NR	All were respondents to a survey about patient and family storytelling at a nursing orientation n(%)<br>n=540(99) agreed that the program provided them with new/valuable information<br>n=506(93) agreed that they were inspired to consider changing something in how they performed their job.
Wiklund Gustin and Wagner, 2013.	N=4.	NR	NR	NR	Clinical nursing teachers	NR	NR	Descriptive paper about the Butterfly effect of Caring, the nursing teachers met for 12 hours of experiential and reflective work.
Brathovde, 2017.	N=24.	Female 95%<br>Male 5%.	Age %<br>21-30 46%<br>31-40 12%<br>41-50 21%<br>51-60 21%.	Caucasian 79%<br>Asian 17%<br>Hispanic 4%.	Baccalaureate prepared nurses (75%) and Masters prepared nurses (25%) from specialty areas such as emergency, medical-surgical, behavioral health, perioperative and nursing administration.	0-5years 54%<br>11-15 years 4%<br>16-20 years 8%<br>26-30 years 25%.	NR	
Mahon et al., 2017.	N=90.	Female n=89<br>Male n=1.	Mean = 44 (SD 8.3) Range 24-62.	NR	Staff nurse n=71(78.9)<br>Clinical specialist n=4(4.4)<br>Nurse manager n=12 (13.3)<br>Midwife n=3 (3.3)	21.1 (SD 9.2)<br>Range 3-44.	NR	Years in current post<br>0-5 18%<br>6-10 30%<br>11-15 26%<br>16-20 9%<br>21+ 18%

Study	Number of participants (N)	Gender n(%)	Age (years)	Race n(%)	Qualification/Job title n(%)	Years in practice	Previous experience related to the program	Other
Richards et al., 2006.	N=12.	Female n=12(100).	Age (years) n(%)<br>20-29 n=2 (17)<br>30-39 n=0 (0)<br>40-49 n=3 (2)<br>50-59 n=5 (42)<br>61 n=2 (17).	NR	Registered nurse (RN) n=7 (58)<br>RN/case manager n=2 (17)<br>Nurse practitioner n=1 (8)<br>Licensed practical nurse n=1 (8)<br>Nursing student/certified medical assistant n=1 (8).	Years of education n(%)<br>14 n=2(17)<br>15 n=1(8)<br>16 n=5(42)<br>17 n=1(8)<br>18 n=3(25).	Previous meditation n (%)<br>Yes n=5(42)<br>No n=7(58).	Spiritual identity n(%)<br>Spiritual and religious n=4(33)<br>Spiritual, but not religious n=7(58)<br>Religious but not spiritual n= 1(8).
**CLINICIANS**								
Primary care								
Karkabi et al., 2014.	N=23.	Female n=13(57)<br>Male n=10 (43).	NR	NR	Family medicine physicians or physicians in training.	NR	NR	Multinational workshop (10 countries).
Verweij et al., 2016.	N=50.	Female n=17 (34)<br>Male n=33(66).	Mean=54.9(SD 5.7).	NR	GPs in training 100%.	Mean duration of practice =24.4(SD6.3) Range 14-39.	NR	Intervention group n=23 Control group n=20. There were no baseline differences between the groups in age, sex, practice setting, and years in practice. However, the intervention group did report significantly more depersonalisation, less work engagement, and fewer mindfulness skills than the control group. They also reported more emotional exhaustion (trend) and less work engagement (trend).
Schroeder et al., 2016.	N=33	Female n=24(73)	Mean=42(SD8.4)<br>Range 32-61.	NR	Physicians 100%.	Mean years licensed	Current meditation practice n=4.	

Study	Number of participants (N)	Gender n(%)	Age (years)	Race n(%)	Qualification/Job title n(%)	Years in practice	Previous experience related to the program	Other
		Male n=9(27)..				=13.3(SD8)<br>Range 3-31.		
Fortney et al., 2013.	N=30.	Female n=18(60)<br>Male n=12(40).	Mean=40.5<br>Range 27-63.	White n=1(97)<br>Hispanic n=1(3).	Nurse practitioner n=1(3)<br>Physician assistant n=3(10)<br>Physician n=26(87).	NR	Mindfulness training in the past n (%)<br>Yes n=2(7) No n= 28(93)<br>Other meditation retreats<br>Yes n=14(47) No n=16 (53)<br>Current regular mediation practice<br>Yes n= 2(7) No n=28(93).	Percentage of work time in clinical practice n (%)<br>50% n=6(20)<br>51%-99% n=19(63)<br>100% n=5(17)<br>Reason for participation<br>Health enhancement n=27(90)<br>Disease management n=3(10)<br>Spiritual growth n=20 (67)<br>Job satisfaction n=21 (70)<br>Curiosity n=15(50)<br>Mindfulness/meditation training or refresher n=19(63)<br>Recommended n=2(7).
Palliative care								
Arnold et al., 2016.	N=75.	NR	NR	NR	All were palliative physicians completing a one-year palliative medicine fellowship.	NR	NR	Retrospective study.
Mental health								
Riches et al., 2019.	N=15 (post intervention).	Female n=14(93)<br>Male n=1(7)	18-25 n=1(7)<br>26-35 n=14(93).	White British n=12(80)<br>White (other) n=2(13)<br>Asian/Asian British n=1(7).	Clinical psychologists n=4<br>Trainee clinical psychologists n=11.	First year trainee n=6(40)<br>Second year trainee n=5(33)<br>Final year trainee n=0<br>Qualified less than 1 year n=4(27).	NR	
**MULTIDISCIPLINARY**								

Study	Number of participants (N)	Gender n(%)	Age (years)	Race n(%)	Qualification/Job title n(%)	Years in practice	Previous experience related to the program	Other
High-risk populations								
Chambliss et al., 1990.	N=14 completed the program.	NR	NR	NR	NR	NR	NR	
Palliative care								
Moore et al., 2017.	N=28 in total<br>n=19 interviewed at 7 months<br>n=19 interviewed at 11 months<br>n=10 interviewed at 15 months.	NR	NR	NR	Nursing home staff and external HCPs.	NR	NR	Residents with advanced dementia recruited for data collection n=9, of these residents' family members were interviewed n=4.
Orellana-Rios et al., 2017.	N=28.	Female n=21(75)<br>Male n=7(25).	Mean=46.4(SD5.8)<br>Range 37-57.	NR	Nurses n=19<br>Physicians n=1<br>Social workers n=2<br>Psychologists n=1<br>Physiotherapists n=1<br>Administration n=3<br>Volunteer n=1.	NR	NR	
Rao and Kemper, 2017.	N=177.	Female n= 148(84)<br>Male n=29(16).	NR	NR	Acupuncturists, chiropractors, massage therapists n=8(5)<br>Dietitians n=5(8)<br>Nurses n=73(41)<br>Physicians n=29(16)<br>Social workers, psychologists, or licensed counsellors n=15(8)<br>Researchers n=5(3)<br>Others, including occupational and physical therapists, laboratory and radiology technicians, unit clerks, volunteers,	NR	NR	n(%)<br>University staff or students n=155(88)<br>Trainees n=30(17).

Study	Number of participants (N)	Gender n(%)	Age (years)	Race n(%)	Qualification/Job title n(%)	Years in practice	Previous experience related to the program	Other
					human resources staff, and others n=32(18).			
Mental health setting								
Suyi et al., 2017.	N=37.	Female n=30(81) Male n=7(19).	Age n(%) Under 25 n=2(5.4) 25–30 n=10(27.0) 30–35 n=9(24.3) 35–40 n=5(13.5) 40–45 n=6(16.2) Over 45 n=5(13.5)	Chinese n=29(78.4) Malay n=2(5.4) Indian n=4(10.8).	Nurse n=8(21.1) Occupational therapist n=3(8.1) Doctor/psychiatrist n=3(8.1) Social worker n=7(18.9) Case manager n=1(2.7) Pharmacist n=1(2.7) Psychologist/counselor n=11(29.7) Researchers n=3(8.1)<br><br>Education Level Diploma n=2(5.4) Degree n=20(54.1) Post-graduate n=14(37.8).	Years of experience Less than 5 n=16(43.2) 6–10 n=6(16.2) 11–20 n=12(32.4) More than 20 n=3 (8.1).	One participant reported practicing a daily 15-minute breath meditation, the others reported no regular meditation practice.	
Elderly care								
Ross et al., 2013.	Human patient simulation training N=93 Ward based training N=86.	NR	NR	NR	Human patient simulation training n=93 Healthcare assistants n=22 Staff nurse n=47 Senior nurse n=15 Doctor n=9 Ward based training n=86 Healthcare assistants n=22 Staff nurse n=44 Senior nurse n=17 Allied Health professional n=3.	NR	NR	

Study	Number of participants (N)	Gender n(%)	Age (years)	Race n(%)	Qualification/Job title n(%)	Years in practice	Previous experience related to the program	Other
Farr and Barker, 2017.	N=22.	Female n=19 (86)<br>Male n=3 (14).	NR	NR	NR	NR	NR	Describes Schwartz Rounds<br>Steering group/coordination role n=4<br>Facilitator/clinical lead role n=7<br>Panelists n=4<br>Attendees n=7<br>Clinical role n=15<br>Senior manager role n=6<br>Non-clinical role n=1.
Gale et al., 2017.	N=10.	Female n=5<br>Male n=5.	Mean=36.2 Range 25-61.	NR	Trainee clinical psychologist n=3<br>Assistant psychologist n=1<br>Specialist Clinical Psychologist n=3<br>Consultant clinical psychologist n=2<br>Psychotherapist n=1.	Trainee/assistants at early career stage n=4<br>Experienced therapists n=6.	All had previously received some Compassion Focused Therapy (CFT) training – the aim of the study was to explore their experiences of personal practice in relation to CFT.	They had undertaken a variety of training in CFT: all had completed a three-day introductory workshop; four had completed advanced training (either an advanced skills workshop or the postgraduate certificate) and two had taken part in a personal practice workshop. Seven participants received CFT-specific supervision.
Mixed settings								
Altamirano-Bustamante et al., 2013.	N=973.	Female 70%<br>Male 30%.	Mean=38.2(SD9.7) Range 17-82. Between 36-50 years 43.5% median=44 years.	NR	Physician 57%<br>Nurse 20%.	NR	Prior training in bioethics 40%.	Level of healthcare<br>First (preventative and family medicine) 37%<br>Second (different medical specialities and general surgery) 35%<br>Third (highly specialised medical

Study	Number of participants (N)	Gender n(%)	Age (years)	Race n(%)	Qualification/Job title n(%)	Years in practice	Previous experience related to the program	Other
								attention) 19%<br>Central 9%<br>Motivation – to improve professional performance (54.6%), increase knowledge of clinical ethics (36.3%).
Reynolds et al., 2019.	Medical students N=219<br>Qualified Healthcare Professionals (HCPs) N=108.	Female 72%<br>Male 28%<br>More HCPs were female (80.6%) than students (68%).	Age range 18-66 years<br>Students were younger (median=22 years) than HCPs (median = 41.5years).	NR	Medical students n=219(67), students were studying medicine (86.3%), nursing (11%) or other clinically relevant specialties (1.8%).<br>Qualified HCPs n=108 of whom<br>Medical doctors 14.4%<br>Nurses 14.4%<br>Other health professionals 4.3%.	HCPs had between 0-46 years of clinical experience (median=12 years), medical students were in their second to sixth year of study (median=4 years).	NR	
Han and Kunik, 2017.	N=24.	Female n=24(100).	Mean=50.75<br>Range =29-73.	NR	Community life/activities n=12(50)<br>Registered nurse/certified nursing assistant n=7(29)<br>Caregiver education n=3(12)<br>Hospice chaplain n=1(4)<br>Exercise physiologist/fitness director n=1(4).	Years of experience in long term care n=16(4-31).	NR	n(%)<br>Use of the program with residents after training n=23(96)<br>Learners who have trained their staff about the program n=20(83).<br><br>Number of staff trained by each learner mean =28 (SD42) Range 0-200.
Penson et al., 2010	Descriptive paper.	NR	NR	NR	NR	NR	NR	Describes Schwartz Rounds.
Kemper and Hill, 2017.	N=22.	Female 96%<br>Male 4%.	Age (%)<br>25-34 21%<br>35-44 33%<br>45-54 21%	Caucasian 83%<br>Hispanic/Latino 4%.	Nurse 71%<br>Physician 8%<br>Nonclinican 8%<br>Other clinical 12%	NR	Aromatherapy 42%<br>Dietary supplements/herbal	Training was by clinician volunteers.

Study	Number of participants (N)	Gender n(%)	Age (years)	Race n(%)	Qualification/Job title n(%)	Years in practice	Previous experience related to the program	Other
			55-64 25%.		Trainee 18%.		remedies 17%<br>Mind-body skills training 38%<br>Massage or reflexology 25%<br>Reiki, Therapeutic Touch, Healing Touch 25%<br>Other (acupressure) 4%<br>None 33%.	
Kemper et al., 2017	N=149.	Female n=119 (80)<br>Male n=30 (20).	Age n(%)<br>18-34 n=30(20)<br>35-54 n=66(44)<br>55-74 n=52(35).	Caucasian 119(80)<br>Hispanic/Latino 5(3.)	Nurses n=56(38)<br>Physicians (MD/DO/PA) n=31(21)<br>Social workers and psychologists n=7(5)<br>Dietitians n=6(4)<br>Researcher n=5(3)<br>Other n=44(30).	Months since registration Mean= 14(SD7).	NR	Modules completed Mean =5 (SD5)<br>n(%)<br>Behavior changes as a result of training<br>Self-care 109 (79)<br>Care of others 97(71).
Moffatt-Bruce et al., 2019	Mindfulness in Motion (MIM) training N=32<br>Flipped classroom resilience training N=50<br>Gabbe Wellness program N=70.	NR	NR	NR	MIM training completed by the entire cardiovascular ICU team (nurses, physicians, respiratory therapists, rehabilitation team members and environmental services personnel.<br>Flipped classroom training completed by emergency medicine, internal medicine and general surgery physicians<br>Gabbe Wellness program completed by nurses, physicians,	NR	NR	

Study	Number of participants (N)	Gender n(%)	Age (years)	Race n(%)	Qualification/Job title n(%)	Years in practice	Previous experience related to the program	Other
					other care providers and staff.			
